# Temperature sensitivity of Notch signaling underlies species-specific developmental plasticity and robustness in amniote brains

**DOI:** 10.1038/s41467-021-27707-5

**Published:** 2022-01-10

**Authors:** Tadashi Nomura, Kohjiro Nagao, Ryo Shirai, Hitoshi Gotoh, Masato Umeda, Katsuhiko Ono

**Affiliations:** 1 Developmental Neurobiology, Kyoto Prefectural University of Medicine, INAMORI Memorial Building, 1-5 Shimogamo-Hangi cho, Sakyo-ku, Kyoto 606-0823 Japan; 2 Department of Synthetic Chemistry and Biological Chemistry, Graduate School of Engineering, Kyoto University, Katsura, Nishikyo-ku, Kyoto 615-8510 Japan; 3 Department of Biophysical Chemistry, Kyoto Pharmaceutical University, 5 Misasaginakauchi-cho, Yamashina-ku, Kyoto 607-8414 Japan; 4 School of Medicine, Niigata University, 757 Ichibancho, Asahimachi-dori, Chuo Ward, Niigata City, 951-8510 Japan; 5 Japanese Red Cross Society Kyoto Daini Hospital, 355-5 Haru-obi cho, Marutamachi- noboru, Kamaza-dori, Kamigyo-ku, Kyoto 602-8026 Japan; 6 HOLO BIO Co., Ltd. 1-36 Goryo Ohara, Nichikyo-ku, Kyoto 615-8245 Japan

**Keywords:** Evolutionary developmental biology, Developmental neurogenesis, Cell signalling, Development of the nervous system

## Abstract

Ambient temperature significantly affects developmental timing in animals. The temperature sensitivity of embryogenesis is generally believed to be a consequence of the thermal dependency of cellular metabolism. However, the adaptive molecular mechanisms that respond to variations in temperature remain unclear. Here, we report species-specific thermal sensitivity of Notch signaling in the developing amniote brain. Transient hypothermic conditions increase canonical Notch activity and reduce neurogenesis in chick neural progenitors. Increased biosynthesis of phosphatidylethanolamine, a major glycerophospholipid components of the plasma membrane, mediates hypothermia-induced Notch activation. Furthermore, the species-specific thermal dependency of Notch signaling is associated with developmental robustness to altered Notch signaling. Our results reveal unique regulatory mechanisms for temperature-dependent neurogenic potentials that underlie developmental and evolutionary adaptations to a range of ambient temperatures in amniotes.

## Abstract (web summary)

Ambient temperature significantly affects embryogenesis, but adaptive molecular mechanisms that respond to temperature remain unclear. Here, the authors identified species-specific thermal sensitivity of Notch signaling in developing amniote brains.

## Introduction

The complex interplay of genes and the environment is responsible for the emergence of constant and variable traits in organisms. Canalization and phenotypic plasticity, which represent developmental robustness and flexibility in response to genetic and environmental perturbations, are thought to play critical roles in the control of phenotypic stability and divergence^1–4^. Understanding the regulatory mechanisms underlying these variable phenotypic outcomes is necessary to clarify the intrinsic forces that spur adaptive evolution in a wide range of environmental conditions.

Ambient temperature significantly influences the developmental programs of both ectothermic and endothermic animals^5–7^. In general, lower temperatures decelerate developmental timing in egg-laying (oviparous) species, which has been assumed to result from the thermal dependency of cellular metabolism^8,9^; for example, departures from the optimum temperatures of biochemical reactions prolong cell divisions and differentiation. However, several species exhibit remarkable plasticity to temperature variations^5^. With only a few exceptions^10–12^, our knowledge of the adaptive molecular mechanisms that underlie the thermal sensitivity and robustness of developmental signaling is still limited.

Notch signaling has been well documented as an important regulator of cell fate decisions during embryogenesis^13^. Canonical Notch signaling is activated by the binding of membrane-bound ligands (Delta, Serrate, and Lag2) to the Notch receptor, which triggers receptor cleavage and the translocation of the Notch intracellular domain (NICD) into the nucleus. Within the nucleus, the NICD forms a transcriptional regulatory complex with other coactivators that controls downstream gene expression. In the developing central nervous system, Notch signaling plays a crucial role in neurogenesis in a variety of species^13,14^: the loss of Notch signaling accelerates neuronal differentiation, while increased Notch signaling preserves neural progenitor pools and suppresses neurogenesis^15,16^. In contrast to the severe phenotypes produced by experimental manipulations, physiological levels of Notch signaling are highly stable to variable temperatures^17^. In *Drosophila*, the net level of Notch signaling is regulated by temperature-dependent compensatory changes in the flux of endocytic trafficking networks^18^. However, the evolutionary conservation and diversification of the thermal sensitivity of Notch signaling and the associated phenotypic influences remain to be elucidated.

Here, we report species-specific thermal dependency of Notch signaling in the neural progenitors of developing amniote brains. Transient hypothermic incubation significantly increases canonical Notch activity in chick neural progenitors, which is mediated by ligand ubiquitylation and internalization. We found that temperature-dependent changes in the biosynthesis and cell surface exposure of phosphatidylethanolamine (PE), a major membrane glycerophospholipid, contribute to the thermal responsiveness of Notch signaling in chick neural progenitors. In contrast, Notch signaling levels are highly stable to temperature variations in developing mouse neocortical progenitors, which provides a safeguard of Notch-dependent neuronal migration during mammalian-specific neocortical development. Our results revealed that the temperature-dependent regulation of Notch signaling is tightly coupled with species-specific neurogenic programs, which underlies multiple levels of developmental canalization in response to environmental changes during amniote evolution.

## Results

### Inverse temperature dependency of Notch signal in nonmammals

**Fig. 1:**
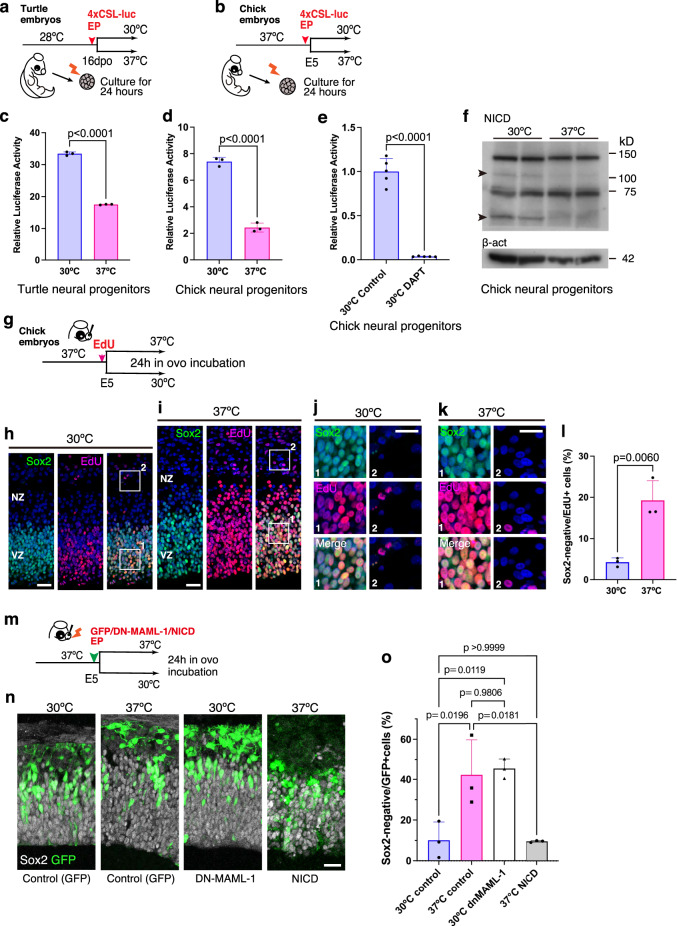
Inverse temperature dependency of Notch activity in neural progenitors of oviparous animals. **a**, **b** Monitoring Notch activities in turtle (**a**) and chick (**b**) neural progenitors cultured at 30 °C or 37 °C for 24 h. A Notch reporter vector (4xCSL-luc) was introduced to isolated neural progenitors prior to culture. **c**, **d** Notch reporter activities in turtle (**c**) and chick (**d**) neural progenitors cultured at 30 °C or 37 °C (mean + SD, *n* = 3 biologically independent samples in each group). **e** DAPT treatment suppressed Notch activity in chicken neural progenitors cultured at 30 °C (means + SD, *n* = 5 biologically independent samples in each group). **f** NICD contents of chick neural progenitors cultured at different temperatures. **g** Schematic illustration of the temperature shift experiment in developing chick embryos. **h**–**k** Distribution of Sox2 and/or EdU-positive cells in the developing chick pallium incubated at 30 °C (**h**, **j**) or 37 °C (**i**, **k**). Insets represent Sox2-positive or negative EdU-labeled cells. **l** The proportion of Sox2-negative and EdU-positive cells in the chick pallium incubated at different temperatures (means + SD, *n* = 3 biologically independent samples in each group). **m** Schematic illustration of DN-MAML1 or NICD overexpression in the developing chick pallium. **n** Distribution of GFP-positive cells in GFP control, DN-MAML1- or NICD-overexpressing chick pallium incubated at 30 °C or 37 °C. **o** The proportion of Sox2-negative and GFP-positive cells in controls (30 °C or 37 °C), and DN-MAML1- or NICD-overexpressing chick pallium at 30 °C or 37 °C (means + SD, *n* = 3 biologically independent samples in each group). Two-sided, unpaired *t*-test for **c**, **d**, **e**, and **l**; ordinary one-way ANOVA for **o** (*p*-values were adjusted by Tukey test for multiple comparisons). *P*-values are indicated in each graph. Scale bars: 25 µm.

To investigate the temperature dependency of Notch activities in developing reptilian and avian brains, we isolated neural progenitors from the pallium of turtle and chick embryos and incubated them for 24 h at 30 °C (an optimal temperature for reptilian embryogenesis, but hypothermic for Aves) or 37 °C (normothermic for avian embryogenesis, but hyperthermic for reptiles). To monitor Notch activity under different temperature conditions, a Notch reporter vector (4xCSL-luc) was introduced into isolated neural progenitors prior to culture (Fig. 1a, b). We found inverse temperature dependence of Notch activity in the two species: culture at 30 °C significantly increased Notch reporter activities in turtle and chick neural progenitors relative to the culture at 37 °C (Fig. 1c, d). The γ-secretase inhibitor DAPT completely blocked Notch activity in chick neural progenitors at 30 °C, suggesting that hypothermia-induced Notch activity is mediated by Notch receptor cleavage (Fig. 1e). Accordingly, the low temperature increased the abundance of cleaved forms of the Notch1 receptor (Notch intracellular domain: NICD) in cultured chick neural progenitors (Fig. 1f). In contrast, Notch activity was lower in chicken liver primary cells, heart muscle cells, and DF-1 fibroblast cells at 30 °C than at 37 °C (Fig. S1a–c). These data suggest that increased Notch activity at lower temperatures is unique to the neural progenitors of oviparous amniotes.

Next, we examined the impact of temperature shifts on pallial neurogenesis in developing chick embryos. Fertilized chicken eggs incubated at 37 °C until E5 (Hamburger and Hamilton stages 24/25) were shifted to 30 °C (hypothermic for chick embryogenesis) for 24 h, when massive neurogenesis occurs in the developing pallium. Neural progenitors residing in the ventricular zone was labeled by administration of a small amount of 5-ethynyl-2’-deoxyuridine (EdU) or the electroporation of GFP expression vector prior to the temperature shift (Figs. 1g and S1i). To examine S-phase re-entry of neural progenitors under different temperatures, ventricular cells were sequentially labeled with GFP expression vector and EdU at different timing (Fig. S1i). Hypothermia significantly delayed the progression of developmental stages of chicken embryos (Fig. S1d–f). Although the number of phosphorylated histone H3-positive mitotic cells in the pallium was comparable (Fig. S1g, h), the proportion of S-phase re-entry among ventricular progenitors was reduced under hypothermic conditions (Fig. S1j, k, m). Furthermore, the number of Sox2-negative cells among the EdU-labeled cells in the pallial neuronal zone was significantly reduced at 30 °C relative to that at 37 °C (Fig. 1h–l). These data suggested that the lower temperature specifically suppressed cell cycle progression and neuronal differentiation in the developing chick pallium.

To determine whether the reduced neuronal differentiation observed in chick embryos under hypothermic conditions was due to the augmented activity of Notch signaling, we tried to block Notch signaling by using a dominant-negative form of Mastermind like transcriptional coactivator 1 (DN-MAML1) that interferes with Notch-dependent transcriptional activity^19^. We electroporated a GFP expression vector either alone or together with a vector expressing DN-MAML1 into the developing chick pallium and incubated the embryos for 24 h under normothermic and hypothermic conditions (Fig. 1m). Consistent with previous results, low temperature significantly reduced the number of GFP-labeled neurons and maintained neuronal progenitors in the ventricular zone, while the hypothermia-dependent reduction in neuronal differentiation was completely restored by the overexpression of DN-MAML1 (Fig. 1n, o). Conversely, the overexpression of NICD at 37 °C significantly reduced neuronal differentiation and cell cycle re-entry in the developing chick pallium (Figs. 1n, o and S1l, m). These data indicate that the thermal sensitivity of Notch signaling underlies the temperature dependency of neurogenesis in the developing chick pallium.

### Endocytosis mediates hypothermia-induced Notch activation

**Fig. 2:**
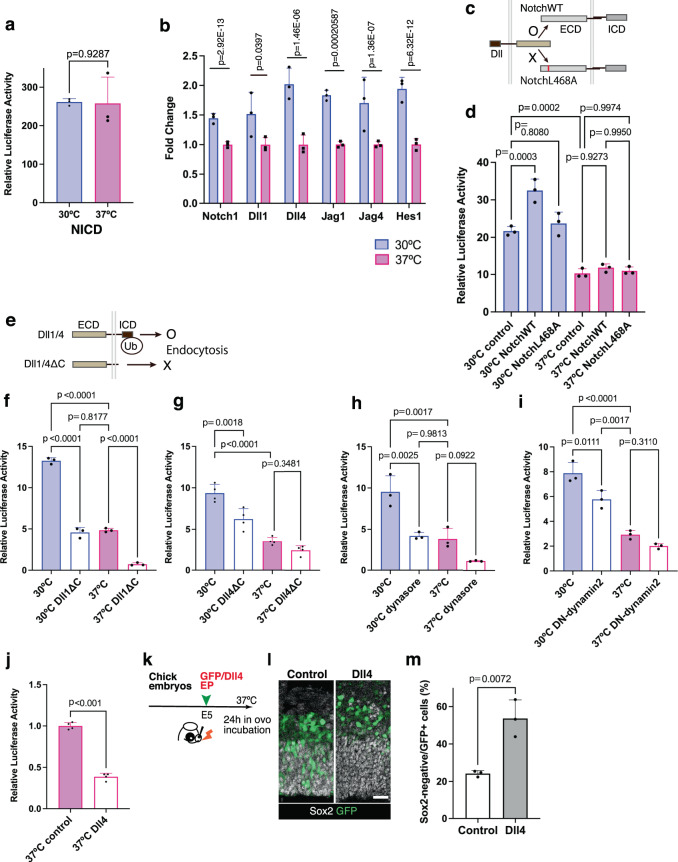
Ligand endocytosis mediates temperature-dependent Notch activity. **a** NICD-dependent Notch reporter activity in chick neural progenitors cultured at 30 °C or 37 °C (mean + SD, *n* = 3 biologically independent samples in each group). **b** Relative expression levels of genes encoding Notch signaling components in chick neural progenitors cultured at 30 °C or 37 °C (mean + SD, *n* = 3 biologically independent samples in each group). **c** Overexpression of Notch1 (NotchWT) or mutant Notch (NotchL468A) receptors in developing chick neural progenitors. **d** Notch reporter activities in control, NotchWT, and NotchL468A overexpressing samples cultured at 30 °C or 37 °C (mean + SD, *n* = 3 biologically independent samples in each group). **e** Schematic drawing of Dll1/4 and Dll1/4ΔC; the latter lacks the intracellular domain (ICD) required for ubiquitylation and endocytosis. **f**, **g** Notch reporter activity in controls and Dll1ΔC- (**f**) or Dll4ΔC (**g**) -overexpressing neural progenitors cultured at 30 °C or 37 °C [mean + SD, *n* = 3 (**f**) and 4 (**g**) biologically independent samples in each group]. **h** Notch reporter activity in control and dynasore-treated neural progenitors cultured at 30 °C or 37 °C (mean + SD, *n* = 3 biologically independent samples in each group). **i** Notch reporter activity in control and dominant-negative dynamin 2 (DN-dynamin 2) overexpressing neural progenitors cultured at 30 °C or 37 °C (mean + SD, *n* = 3 biologically independent samples in each group). **j** Notch reporter activity in control and Dll4-overexpressing neural progenitors cultured at 37 °C (mean + SD, *n* = 4 biologically independent samples in each group). **k** Schematic illustration of GFP or Dll4 overexpression in the developing chick pallium. **I** Distribution of Sox2 and/or GFP-positive cells in the developing chick pallium in control and Dll4 electroporated samples. **m** The proportion of Sox2-negative and GFP-positive cells in controls and Dll4-overexpressing chick pallium at 37 °C (means + SD, *n* = 3 biologically independent samples in each group). Two-sided, unpaired *t*-test for **a**, **j**, and **m** (*p*-values are indicated in each graph), two-sided, negative bimodal distribution for **b** (*p*-values were adjusted for multiple comparisons by using Benjamin–Hochberg method, FDR of Dll1 is not true), ordinary one-way ANOVA for **d** and **f**–**i** (*p*-values were adjusted by Tukey test for multiple comparisons). Scale bar: 25 µm.

We explored the potential mechanisms underlying the inverse temperature dependency of Notch activation in developing chick neural progenitors. Notch activity induced by the overexpression of NICD did not differ between normothermic and hypothermic conditions (Fig. 2a), indicating that NICD-dependent transcriptional regulation is not temperature-sensitive. We also examined the expression levels of genes encoding secretase components that are responsible for Notch receptor cleavage. RNA-seq analysis indicated that the expression of *ADAM metallopeptidase domain 10* (*ADAM10*) and *Presenilin1* (*PSEN1*), major components of α-and γ-secretases, respectively, were considerably lower in neural progenitors cultured at 30 °C than in those cultured at 37 °C (Fig. S2a), suggesting that hypothermia-induced Notch activation is not due to increased enzymatic activities related to receptor processing. Next, we examined the expression profiles of mRNAs encoding the Notch signaling components in cultured neural progenitors. Hypothermia significantly increased the expression levels of genes encoding Notch receptor (*Notch1*) and ligands, such as *Delta like 4* (*Dll4*), *Jagged 1(Jag1)*, and *Jagged 4* (*Jag4*), as well as the downstream target gene *Hes1* (Fig. 2b). To determine whether hypothermia-induced Notch activation is due to increased receptor expression, we overexpressed the Notch1 receptor in chick neural progenitors. The introduction of the Notch1 receptor did not increase Notch activity under normothermic conditions, while it further increased hypothermia-dependent Notch activity under hypothermic conditions. In contrast, a mutant Notch1 receptor (NotchL468A) that was unable to bind ligands^20^ did not facilitate Notch activity under either normothermic or hypothermic conditions (Fig. 2c, d). Thus, although the increase in Notch receptor expression was not sufficient to reproduce hypothermia-induced Notch activation, ligand-dependent Notch receptor activity was facilitated by a lower temperature in developing chicken neural progenitors. To further confirm the requirement of endogenous ligands for temperature-dependent Notch activity, we introduced a synthetic Notch receptor (SynNotch) that was designed to reproduce Notch-dependent cellular reactions in response to artificial ligands^21^. Although the ligand-dependent activation of SynNotch signaling was recapitulated in cultured chicken neural progenitors, we could not detect a hypothermia-dependent increase in reporter activity with an artificial ligand (Fig. S2b), suggesting that the temperature sensitivity of Notch signaling depends on the properties of the endogenous ligands in developing chicken neural progenitors.

To investigate the distribution of endogenous ligands in chicken neural progenitors under different temperature conditions, we determined the cellular localization of the Dll ligand by a cell surface biotinylation assay. Under hypothermic conditions, the relative amount of the Dll ligand on cell surface was decreased, while it was enriched in the intracellular fractions, suggesting that lower temperature increased ligand internalization, rather than cell surface representation (Fig. S2c,d). Accordingly, hypothermia facilitated the ubiquitylation of the Dll ligand (Fig. S2e), which is essential for ligand internalization. Furthermore, the overexpression of Dll1 or Dll4 lacking the intracellular domain responsible for ubiquitylation (Dll1ΔC or Dll4ΔC) suppressed hypothermia-induced Notch activity in cultured chick neural progenitors (Fig. 2f, g), corroborating the requirement of ligand ubiquitylation and internalization for hypothermia-dependent Notch activation.

Several studies have shown that the endocytosis of Notch ligands is a prerequisite for Notch signaling activation^22^. We confirmed that the pharmacological blockade of dynamin-dependent endocytosis significantly suppressed temperature-dependent Notch activity in chicken neural progenitors (Fig. 2h). Furthermore, hypothermia-induced Notch activity was reduced by dominant-negative dynamin 2 (Fig. 2i), but not by dominant-negative dynamin 1 (Fig. S2f), suggesting that dynamin 2-dependent ligand endocytosis^23^ is critical for the enhancement of Notch activity in developing chicken neural progenitors under hypothermic conditions.

To examine whether the increased expression of Notch ligands is sufficient to activate Notch signaling, we overexpressed Dll4 in chick neural progenitors. However, relative to the controls, the introduction of Dll4 decreased Notch activity at 37 °C (Fig. 2j). We also confirmed that the electroporation of Dll4 increased the proportion of Sox2-negative cells in the developing chick pallium (Fig. 2k–m), in accord with the previous finding that Dll ligands reduce Notch activity and facilitate neuronal differentiation^24^. These data suggest that the increased expression of Notch ligands is not sufficient to increase Notch activity, and additional mechanisms might underlie the temperature sensitivity of Notch signaling in developing chick neural progenitors.

### Hypothermia increases specific phospholipids in chick

**Fig. 3:**
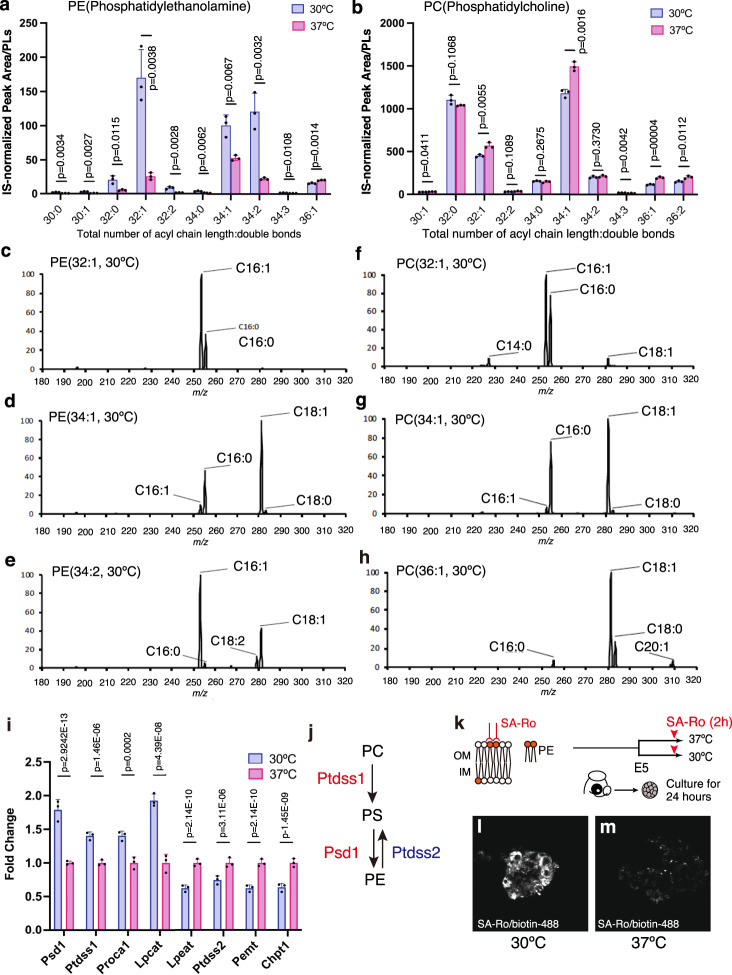
Enrichment of cell surface PE mediates Notch signaling. **a**, **b** PE (**a**) and PC (**b**) molecule contents of chick neural progenitors cultured at 30 °C or 37 °C (mean + SD, *n* = 3 biologically independent samples in each group). **c**–**h** Fatty acid composition of PE (32:1, **c**; 34:1, **d**; 34:2, **e**) or PC (32:1, **f**; 34:1, **g**; 36:1, **h**) in chick neural progenitors cultured at 30 °C. **i** Relative expression levels of enzymes involved in PE synthesis in chick neural progenitors cultured at 30 °C or 37 °C (mean + SD, *n* = 3 biologically independent samples in each group). **j** Biosynthesis of PE through the Psd pathway. **k** Labeling of cell surface PE by SA-Ro treatment in nonpermeable condition. **l**, **m** SA-Ro fluorescent labeling of chick neural progenitors cultured at 30 °C (**l**) or 37 °C (**m**). Two-sided, unpaired *t*-test for **a**, **b**, two-sided, negative bimodal distribution for **i** (*p*-values were adjusted for multiple comparisons by using Benjamin–Hochberg method). *P*-values are indicated in each graph.

To reveal the potential mechanisms underlying the inverse temperature dependency of Notch activity in chick neural progenitors, we sought to determine the contribution of other biological pathways under hypothermic conditions. Hypothermia-induced global changes in transcriptome components involved in multiple metabolic pathways in cultured chick neural progenitors (Fig. S3a, b). We focused on the enrichment of cellular components associated with the membrane-enclosed lumen in response to the temperature change (Fig. S3a). Several studies have shown that temperature-dependent changes in membrane lipid compositions confer thermal adaptability by modulating the physical properties of plasma membranes and membrane-associated cellular events^25–28^. Accordingly, we performed mass spectrometry to compare the major phospholipid components of chicken neural progenitors under normothermic and hypothermic culture conditions. Notably, hypothermic culture increased specific molecular species of phosphatidylethanolamine (PE) containing shorter acyl chains and fewer double bonds (Figs. 3a and S4a). Product ion scan analysis by LC-MS/MS showed that most of the PE species affected by hypothermia consisted of C16–18 saturated or unsaturated fatty acids with one double bond (Fig. 3c–e). Conversely, the abundance of specific species of phosphatidylcholine with similar fatty acid characteristics to the increased PE species was reduced by hypothermic culture conditions (Figs. 3b, f–h and S4b), suggesting that hypothermia induced the de novo synthesis of PE at the expense of PC production.

There are two major metabolic pathways involved in PE biosynthesis: the CDP-ethanolamine pathway and the phosphatidylserine decarboxylase (Psd) pathway^29^. The RNA-seq analysis of chicken neural progenitors identified hypothermia-dependent increases in *phosphatidylserine decarboxylase 1* (*Psd1*) and *phosphatidylserine synthase 1* (*Ptdss1*) but a decrease in *Ptdss2*, suggesting that hypothermia facilitated the directional biosynthesis of PE through the Psd pathway (Fig. 3i, j). In contrast, we could not detect altered expression of genes associated with the CDP-ethanolamine pathway, such as *ethanolaminphosphotransferase 1* (*EPT1L*; deposited to Mendeley Data: DOI: 10.17632/9zxt47grjf.2).

To examine the cellular distribution of PE in developing chicken neural progenitors, we utilized a streptavidin-conjugated peptide probe (SA-Ro) that specifically binds to PE^30^. PE is usually enriched on the inner leaflet of plasma membranes, while it is exposed to cell surfaces during specific cellular events^30–33^, which can be detected by using a peptide probe under nonpermeabilized conditions (Fig. 3k). We found that hypothermia dramatically increased the SA-Ro-mediated fluorescent labeling of chicken neural progenitors (Fig. 3l, m), suggesting that an increased amount of PE was exposed on the cell surface under hypothermic conditions. Temperature-dependent changes in PE and PC contents were also evident in cultured turtle neural progenitors, although the impact of temperature variations was much weaker than that in chick neural progenitors (Fig. S4c, d). Thus, the thermal sensitivity of phospholipid synthesis might be shared among oviparous amniotes.

### Enrichment of PE affects Notch signal in chick progenitors

**Fig. 4:**
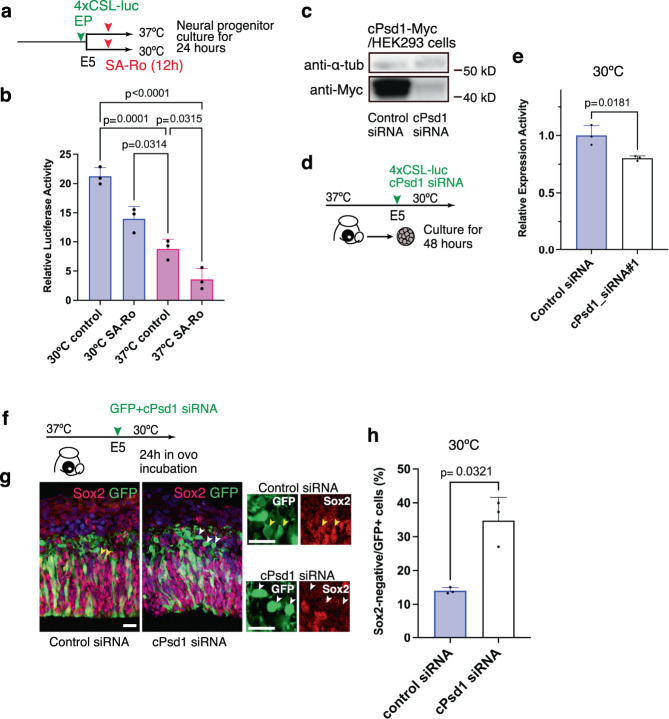
PE mediates hypothermia-dependent increases in Notch activity. **a** Trapping of cell surface PE in cultured chick neural progenitors by treatment with SA-Ro for 12 h. **b** Notch reporter activity in chick neural progenitors treated with SA-Ro at different temperatures (mean + SD, *n* = 3 biologically independent samples in each group). **c** Western blotting of Myc-tagged chick Psd1 in HEK293T cells transfected with siRNAs. **d** Introduction of the Notch reporter vector and siRNA targeting *Psd1* in isolated chick neural progenitors. **e** Notch reporter activity in chick neural progenitors transfected with control or *Psd1* siRNAs (mean + SD, *n* = 3 biologically independent samples in each group). **f** Schematic illustration of *cPsd1* siRNA introduction in the developing chick pallium. **g** Distributions of GFP-positive cells in control and *cPsd1* siRNA overexpressing chick pallium incubated at 30 °C. **h** The proportion of Sox2-negative and GFP-positive cells in control and *cPsd1* siRNA overexpressing chick pallium at 30 °C (means + SD, *n* = 3 biologically independent samples in each group). Ordinary one-way ANOVA for **b** (*p*-values were adjusted by Tukey test for multiple comparisons), two-sided, unpaired *t*-test for **e**, **h**. *P*-values are indicated in each graph. Scale bars: 25 µm.

To examine the functional contribution of PE to Notch signaling, we treated chick neural progenitors with SA-Ro for 12 h to trap cell surface PE and examined Notch reporter activity (Fig. 4a). SA-Ro application significantly reduced Notch activity in neural progenitors under both normothermic and hypothermic conditions (Fig. 4b). To further confirm the role of PE in Notch signaling, we designed an siRNA that downregulates *Psd1*, a gene responsible for PE synthesis. The knockdown of chick *Psd1* using siRNA significantly decreased Notch reporter activity in chick neural progenitors under hypothermic conditions (Fig. 4c–e). These results indicate that hypothermia-dependent activation of Notch signaling is mediated by increased PE synthesis and cell surface exposure in chick neural progenitors. To determine whether altered PE synthesis affects neuronal differentiation, we introduced siRNA targeting chick *Psd1* together with a GFP expression vector into the developing chick pallium. Compared to the results in the controls, the electroporation of *Psd1* siRNA significantly increased the proportion of Sox2-negative cells delaminated from the ventricular zone (Fig. 4f–h), suggesting that reduced PE synthesis in ventricular progenitors increased neuronal differentiation. Taken together, these data indicate that temperature-dependent changes in PE synthesis play a significant role in the regulation of neurogenesis by modifying Notch signaling in the developing chick brain.

### Notch signal is stable to temperature shifts in mouse

**Fig. 5:**
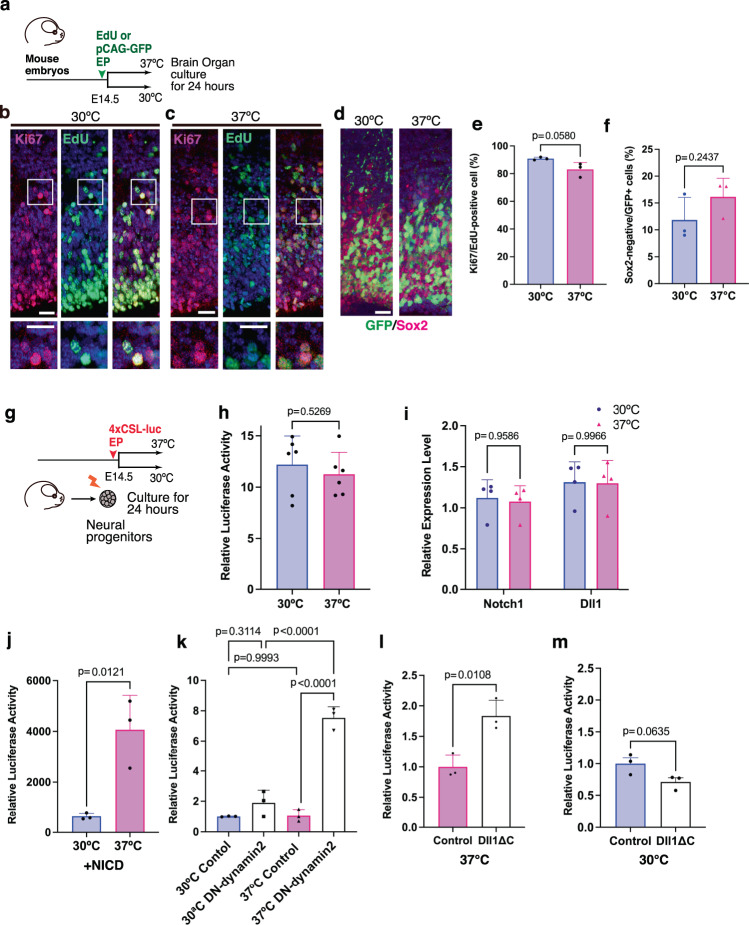
Temperature robustness of Notch signaling in mouse neural progenitors. **a** Schematic illustration of the temperature shift experiment conducted on the developing mouse brain. **b**, **c** Distribution of Ki67-positive and/or EdU-positive cells in the developing mouse neocortex cultured at 30 °C (**b**) or 37 °C (**c**). **d** Distribution of GFP-positive cells in the developing mouse neocortex cultured at 30 °C or 37 °C. **e**, **f** The proportion of Ki67/EdU-positive cells (**e**) and Sox2-negative/GFP-positive cells (**f**) in the developing mouse neocortex cultured at 30 °C or 37 °C (mean + SD, *n* = 3 biologically independent samples in each group). **g** Monitoring of Notch activity in mouse neocortical neural progenitors cultured at 30 °C or 37 °C for 24 h. **h** Notch reporter activities in mouse neural progenitors cultured at 30 °C or 37 °C (means + SD, *n* = 6 biologically independent samples in each group). **i** Relative expression levels of genes encoding *Notch1* and *Dll1* in mouse neural progenitors cultured at 30 °C or 37 °C (means + SD, *n* = 4 biologically independent samples in each group). **j** NICD-dependent Notch reporter activities in mouse neural progenitors cultured at 30 °C or 37 °C (means + SD, *n* = 3 biologically independent samples in each group). **k** Notch reporter activities in control and dominant-negative dynamin 2 (DN-dynamin 2)-overexpressing neural progenitors cultured at 30 °C or 37 °C (means + SD, *n* = 3 biologically independent samples in each group). **I**, **m** Notch reporter activity in control neural progenitors and neural progenitors overexpressing Dll1 lacking intracellular domain (Dll1ΔC) cultured at 37 °C or 30 °C (mean + SD, *n* = 3 biologically independent samples in each group). Two-sided, unpaired *t*-test (**e**, **f**, **h**, **j**, **l**, **m**), ordinary one-way or two-way ANOVA for **k** and **i** (*p*-values were adjusted by Tukey or Sidak test for multiple comparisons). Scale bars: 20 µm.

To further explore the evolutionary conservation of temperature-dependent neurogenic controls among amniotes, we investigated the thermal sensitivity of neurogenesis in the developing mammalian neocortex. Thus, we isolated mouse E14.5 brains and performed organotypic culture under normothermic (37 °C) and hypothermic (30 °C) conditions for 24 h (Fig. 5a). Neocortical neural progenitors were labeled by administration of EdU or electroporation of a GFP expression vector prior to the organotypic culture. At 24 h after the temperature shift, we examined cell cycle re-entry and neuronal differentiation in the neocortex cultured under different temperature conditions. Notably, the proportions of Ki67-positive or Sox2-negative cells in the progeny of labeled progenitors were comparable between normothermic and hypothermic conditions (Fig. 5b–f), suggesting that hypothermia did not alter the rate of cell cycle re-entry and neuronal differentiation in the cultured mouse neocortex. Furthermore, cultured neural progenitors isolated from the neocortex exhibited stable Notch activity irrespective of the temperature (Fig. 5g, h). Accordingly, the expression levels of *Notch1* and *Dll1* in mouse neural progenitors did not differ between normothermic and hypothermic conditions (Fig. 5i). These data indicate that Notch signaling in the developing mouse neocortex is robust to a range of temperatures. To investigate potential mechanisms for stabilizing Notch activity under different temperatures, we introduced several constructs that modulate Notch signaling and compared the outcomes under normothermic and hypothermic conditions. We confirmed that NICD-dependent Notch activity was temperature-sensitive and was increased by temperature (Fig. 5j). Next, we investigated the temperature sensitivity of ligand-dependent endocytic pathways in mouse neocortical progenitors. The introduction of DN-dynamin 2 significantly increased Notch activity at 37 °C, suggesting that endocytosis negatively regulates Notch signaling in mouse neural progenitors, in contrast to the finding in chick neural progenitors. (Fig. 5k). Notch activity was also increased by Dll1ΔC in mouse neural progenitors at 37 °C (Fig. 5l), corroborating that ligand-mediated endocytosis negatively controls Notch signaling. Notably, the introduction of DN-dynamin 2 or Dll1ΔC did not facilitate Notch activity under hypothermic conditions (Fig. 5k, m), indicating that the endocytosis-dependent downregulation of Notch activity is also temperature-sensitive. These data suggest that the temperature-dependent transcriptional regulation of Notch signaling is counterbalanced by endocytosis-mediated negative regulation. In contrast to chick neural progenitors, we could not detect notable differences in phospholipid contents in mouse neural progenitors between normothermic and hypothermic conditions (Fig. S5a, b). Accordingly, neither the up- nor downregulation of Psd1 altered neuronal differentiation in the developing mouse neocortex (Fig. S5c–f). Thus, the thermal robustness of Notch signaling is not associated with temperature-dependent lipid synthesis in developing mouse neocortical progenitors.

### Species-specific developmental responses to Notch alteration

**Fig. 6:**
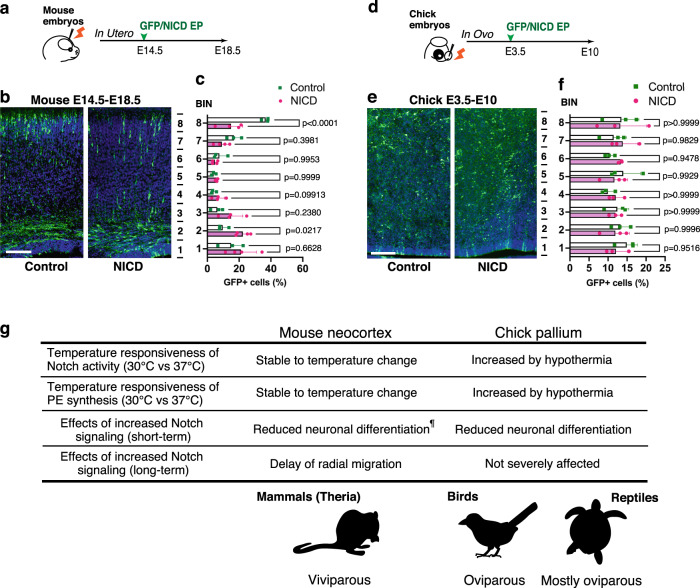
Species-specific susceptibility to altered Notch signaling in amniote pallium. **a**–**f** Transient NICD overexpression in the developing mouse neocortex (**a**–**c**) and chick pallium (**d**–**f**) by *in utero* or *in ovo* electroporation. **a** Schematic illustration of *in utero* electroporation. **b**, **c** Distribution of GFP-positive neurons in the developing mouse neocortex electroporated with the control or NICD expression vector (mean + SD, *n* = 3 biologically independent samples in each group). **d** Schematic illustration of the *in ovo* electroporation experiment. **e**, **f** Distribution of GFP-positive neurons in the developing chick dorsal pallium electroporated with control or NICD expression vector (mean + SD, *n* = 3 biologically independent samples in each group). **g** Summary of the species-specific temperature responsiveness of Notch signaling, PE synthesis, and the effects of increased Notch signaling in the developing amniote pallium (^¶^Refs. ^16,38^). Two-way ANOVA for **c**, **f** (*p*-values were adjusted by Tukey test). Scale bars: 100 µm.

In contrast to oviparous embryos, which are influenced by ambient temperatures, mammalian embryogenesis proceeds within the uterus under stable thermal conditions, raising the question of the biological significance of the thermal robustness of Notch signaling in the developing mammalian brain. One possible interpretation is that the species-specific thermal dependency of Notch signaling is associated with differential susceptibility of developmental programs to altered Notch signaling. We have previously identified species differences in pallial neurogenic patterns and the regulation of developmental signaling in amniotes^34–37^. Mammalian corticogenesis proceeds in an inside-out manner, in which excitatory neurons born in the ventricular zone radially migrate toward the surface of the cortex and adopt a position in a specific cortical layer. In contrast, reptilian and avian palliogenesis proceed in a roughly outside-in manner, in which newly born neurons do not undergo extensive radial migration but intermingle with previously born neurons. Thus, it is possible that stabilizing Notch signaling in mammalian neocortical development is necessary to avoid developmental abnormalities caused by Notch signaling perturbation. To test this possibility, we overexpressed an NICD expression vector in different amniote species (mice, chickens, and turtles) and examined long-term effects on neuronal distribution after electroporation. We labeled neural progenitors when massive neurogenesis occurs in the pallium depending on higher Notch activity, then fixed the embryo at the end of neurogenesis in each species^34^. Accordingly, we electroporated the NICD expression vector into the E14.5 mouse neocortex (Fig. 6a). As previously reported^38,39^, NICD overexpression suppressed neuronal differentiation in the developing mouse neocortex, which resulted in the delay of radial migration and the accumulation of transfected cells in the ventricular and subventricular zones (Fig. 6b, c). Abnormal cell accumulation in the subventricular zone was not fully restored in the postnatal mouse neocortex (Fig. S6a–c), indicating that the effect of altered Notch signaling was not fully compensated. Next, we addressed the effect of the transient activation of Notch signaling in the developing chicken pallium. To precisely compare homologous structures of the neocortex in the chick pallium, we targeted the dorsal pallium of early embryonic stages (E3.5, HH20–22) and examined the cellular distribution at the end of neurogenesis (E10 or E11, Fig. 6d). The electroporation of the NICD expression vector significantly increased the activity of the Hes1 promoter in the developing chick pallium (Fig. S6d–k), corroborating that Notch-dependent downstream regulation is shared among amniotes. In contrast to the finding in the mouse neocortex, however, we could not detect obvious alterations in the cell distribution in the chick pallium after transient Notch activation (Fig. 6e, f). In both controls and NICD transfected embryos, GFP-positive cells were dispersed from deep to superficial parts of the chick dorsal pallium (Fig. 6f), indicating that the effect of altered Notch signaling was restored in the developing chick pallium. Similarly, the siRNA-mediated knockdown of *Psd1* did not affect the cell distribution in the developing chick pallium (Fig. S6l–n). We also confirmed that the neuronal distribution in the developing turtle pallium was not affected by transient NICD overexpression (Fig. S6o–r). These results suggest that mammalian neocortical development is vulnerable to the transient perturbation of Notch signaling, while the developmental programs of the reptilian and avian pallium retain the buffering potential to stabilize phenotypes in response to Notch-dependent changes during embryogenesis. Thus, the thermal robustness of Notch signaling in mammalian neural progenitors might provide a developmental safeguard to prevent deleterious phenotypic consequences in the developing mammalian neocortex.

## Discussion

The species-specific thermal sensitivity of developmental signaling plays a crucial role in the establishment of phenotypic plasticity and robustness in response to temperature variations. Here, we report temperature-dependent changes in neurogenesis in developing reptilian and avian brains, which are mediated by the thermal sensitivity of Notch signaling. Intriguingly, Notch activity is increased by a lower temperature (30 °C), corresponding to hypothermic conditions for chick embryos but normothermia for turtles, indicating that the thermal responsiveness of Notch signaling is shared among oviparous amniotes regardless of species differences in optimal temperatures for embryogenesis. In contrast, Notch activity in developing mouse neural progenitors is highly stable to temperature variations, indicating that differential regulatory mechanisms underlie temperature-dependent regulation of Notch activity between oviparous and viviparous amniotes, despite the extensive conservation of Notch signaling components among species. Increased levels of NICD in the developing chick pallium reduced cell cycle re-entry, corroborating that the hypothermia-dependent inhibition of cell cycle progression is mediated by elevated Notch signaling, as previously reported^40,41^. It is possible that S-phase, during which DNA synthesis occurs, is more sensitive to temperature changes than the mitotic phase in developing neural progenitors.

We found that dynamin-dependent ligand endocytosis is critical for the hypothermia-dependent activation of Notch signaling in developing chick neural progenitors. Several studies have shown that the ubiquitylation and internalization of Notch ligands play a key role in Notch signaling, by producing a “pulling force” that promotes receptor cleavage and/or promotes the recycling of ligands on the cell surface^42^. Accordingly, the lower temperature increased endogenous ligand-dependent Notch activation in chick neural progenitors, suggesting that endocytosis positively regulates hypothermia-induced Notch activation by facilitating the receptor-ligand interaction between juxtaposed cells, or the recycling of ligands in cells sending out signals. In *Drosophila*, lower temperatures reduce ligand-dependent Notch activation, which is compensated by ligand-independent endocytic pathways^18^. In contrast to the finding in chicks, we found that endocytosis negatively regulates Notch signaling in developing mouse neural progenitors, possibly by facilitating degradation pathways of Notch components. Thus, the divergent functions of endocytosis in Notch signaling underlie the species-specific thermal sensitivity of Notch signaling, although further investigations are required to reveal the detailed molecular mechanisms counterbalancing the positive and negative regulation of Notch activation in response to temperature.

We found that a hypothermia-dependent increase in PE contributes to increased Notch activity in developing chick neural progenitors. PE is a major glycerophospholipid that is enriched in the inner leaflet of plasma membranes and significantly affects the fluidity of lipid bilayers, as well as the topology of membrane-associated proteins^29,43^. Furthermore, the exposure of PE on the cell surface contributes to specific cellular events, such as cell mitosis^32^ or cell fusion^33^. We speculate that the enrichment of PE at the cell surface facilitates Notch signaling at multiple levels, including changes in the distribution and conformation of Notch receptors and/or ligands, as well as ligand internalization and recycling to the plasma membrane. A recent study reported that the N-terminus of Notch ligands functions as a phospholipid recognition domain and mediates Notch activation^44^, also suggesting that excess cell surface PE promotes Notch signaling by facilitating ligand-dependent cell-cell interactions under hypothermic conditions.

Although temperature-dependent Notch activation is shared among oviparous amniote species, the profound increase in PE synthesis observed in developing chick neural progenitors is unique to these cells, implying that the temperature sensitivity of Notch signaling is mediated by variable regulatory mechanisms depending on the species-specific cellular metabolism. We found that the major molecular species of chicken PE are composed of monounsaturated fatty acids, while mouse and turtle PE contain polyunsaturated fatty acids. The former can be produced by de novo synthesis through intracellular metabolic pathways, but the latter is derived from external supplements^45,46^. Thus, species-specific differences in lipid metabolism are a key factor conferring phenotypic plasticity and robustness in variable environments, in addition to underlying cold acclimation^47^ and longevity^48,49^, as a result of adaptive evolution in a variety of species.

The transient overexpression of Notch signaling components results in long-term impacts on the cell distribution in the mammalian neocortex but not in the pallium of nonmammalian amniotes. We have previously shown species-specific patterns of neuronal migration in the developing amniote pallium, among which the sequential production of excitatory neuron subtypes and the locomotive mode of neuronal migration are unique characteristics of the developing mammalian neocortex^37^. It has also been reported that NICD controls the radial migration of cortical neurons downstream of Reelin signaling^39^, which is greatly amplified in the developing mammalian neocortex^50^. Thus, we suggest that the temperature robustness of Notch signaling is crucial for canalizing cytoarchitectonic phenotypes minimizing developmental abnormalities in the mammalian neocortex. In contrast, pallial development in reptiles and birds exhibits phenotypic robustness to the acceleration or deceleration of neuronal differentiation depending on Notch signaling, which might underlie the evolutionary adaptation of oviparous amniotes in response to variable environmental temperatures (Fig. 6g). Indeed, temperature fluctuations during embryogenesis are common in many ectothermic animals, and are inversely correlated with the total length of the developmental period depending on the variable climate^51^. Conversely, temperature perturbations also increase the risk of embryonic mortality and developmental malformation in both oviparous and viviparous animals^6^. We found that hypothermia induced global changes in transcriptome components related to a wide range of cellular events, raising further questions regarding the physiological significance of other metabolic pathways in temperature-dependent phenotypic variations, which is a critical issue that remains to be elucidated in future studies.

## Methods

### Animals

Fertilized chicken eggs (*Gallus gallus*) were obtained from a local farm (Yamagishi, Japan) and incubated at 37.0 ± 0.2 °C until the temperature shift. Embryonic stages were determined according to Hamburger-Hamilton stages^52^. At E5 (HH stages 24–25), a group of eggs was transferred to 30.0 ± 0.2 °C and incubated for 24 h. Fertilized eggs of Chinese softshell turtles (*Pelodiscus sinensis*) were obtained from a local farm (Daiwa Yoshoku, Japan) and incubated at 28 ± 0.5 °C. The embryonic stages of the turtles were determined according to developmental stages provided in a previous report^53^. Pregnant female mice [*Mus musculus*, Crl:CD-1 (ICR), 3 months] were obtained from Japan SLC, Inc. The day on which vaginal plugs were found at midday was considered embryonic Day 0.5 (E0.5). Total 17 pregnant mice were used in this study. Diets for mice (MF, Oriental Yeast Co., Ltd.) were purchased from Shimizu Laboratories Supplies Co., Ltd. Housing conditions of mice were as follows: 12 h dark/light cycle, 25 °C, and 60% humidity. All experimental procedures conducted in this study were approved by the experimental animal committee of Kyoto Prefectural University of Medicine and Institutional Animal Care and they were performed in accordance with the relevant guidelines (M2020–193, M2020–4).

### EdU pulse-labeling

The EdU labeling of neurons in the chick pallium and mouse neocortex was performed according to a previous study^34^. Briefly, ~0.05 µL of EdU (Thermo Fisher Scientific, 10 mg mL^−1^) was injected into the lateral ventricle of each chick embryo (HH stages 24–25), and the eggs were incubated at 30 °C or 37 °C for 24 h. To examine EdU-labeled cells were detected with the Click-iT EdU Detection System (Thermo Fisher Scientific).

### Plasmids

The following expression vectors were used in the present study: p4xCSL-firefly luciferase (Addgene #41726), pCAGGS-NICD (Addgene #26891), DN-dynamin 1 (Dynamin 1 K44A, a gift from Dr. Sandra Schmid), DN-dynamin 2 (GFP-dynamin 2 K44A, Addgene #22301), HA-Ubiquitin (Addgene #18712), pHR_SFFV_LaG17_synNotch_TetRVP64 (Addgene #79128), pHR_EGFPligand (Addgene #79129), pCS2-Notch1 FL-6MT (Addgene #41728), pCMV-mouse Dll1 (OriGene), pCMV-mouse Dll4 (OriGene), p6872 pHAGE-N-V5-MAML1-FL (Addgene #37048), and pHes1-GFPd2 (Addgene #14808). pCS2-Notch1 L468A was constructed by site-directed mutagenesis using the PrimeSTAR Mutagenesis Basal Kit (Takara). DN-MAML1 lacking the NICD-binding domain (13L-74H) and Dll1ΔC and Dll4ΔC lacking the intracellular domain (Dll1:569V-728L, Dll4:552A-686V) were amplified by polymerase chain reaction (PCR) and subcloned into pCAG-RB using the In-Fusion HD Cloning Kit (Clontech). pCMV-HA-Notch1 and pCMV-Myc-Ubiquitin were constructed by subcloning the cDNAs of Notch1 or Ubiquitin into pCMV-HA-N or pCMV-Myc-N (Clontech), respectively. Mouse and chick Psd1 cDNAs (ENSMUSG00000023452 and ENSGALT00000011126.6, respectively) were chemically synthesized and subcloned into the pGL3-promoter or pCMV-Myc-N vector.

### Luciferase reporter assay

To quantify Notch signaling activity, p4xCSL-firefly luciferase and pRL-SV40 vectors (Promega) were coelectroporated into dissociated neural progenitors using electroporation cuvettes (SE-202, BEX) and a pulse generator (CUY21-EDIT, BEX). After electroporation, the neural progenitors were cultured as floating cell aggregates in a neurobasal medium supplemented with GlutaMAX, the B27 supplement (Thermo Fisher Scientific) and FGF2 (Thermo Fisher Scientific, 10 ng/mL), as previously described^54^. Chicken primary liver cells and heart muscle cells were isolated from E10 chick embryos and dissociated with 0.25% trypsin. Chicken fibroblast cells (DF-1) were purchased from the ATCC (CRL-12203). These cells were cultured at 30 ± 0.2 °C or 37 ± 0.2 °C for 24 h in incubators supplied with 5% CO_2_. To monitor Notch activity in these cell types, p4xCSL-firefly and pRL-SV40 were transfected into the cells by using Lipofectamine 2000 (Thermo Fisher Scientific), and the cells were cultured in Dulbecco’s modified Eagle’s medium (DMEM) supplemented with fetal bovine serum. To block γ-secretase activity or dynamin-dependent endocytosis, DAPT (Sigma, 10 µM) or Dynasore (Abcam, 50 µM) was added to the culture medium for 24 h. Luciferase activity was examined with the Dual-Luciferase Reporter Assay System (Promega). Chemical luminescence was analyzed with a luminometer (GENE LIGHT GL210A, Microtec). All firefly luciferase values were normalized to Renilla luciferase activities to quantify relative luciferase units. To detect firefly luciferase activity driven by the SynNotch system, HEK293T cells were purchased from RIKEN Bioresource Center (RBRC-RCB2202) and transfected with pHR_SFFV_LaG17_synNotch_TetRVP64 and cultured alone or mixed with neural progenitors transfected with pHR_EGFPligand. Each experiment was carried out with at least three biologically independent samples.

### Immunohistochemistry

Brains were fixed with 4% paraformaldehyde dissolved in phosphate-buffered saline (PBS) at 4 °C overnight. After washing with PBS, the brains were cryoprotected with a 30% sucrose solution and immersed in Tissue-Tek. The frozen brains were sectioned at a thickness of 20 µm using a Cryostat (Leica CM1850, Germany), and incubated with primary antibodies, including anti-Sox2 (Abcam, ab97959, rabbit polyclonal,1:1000), anti-phosphorylated histone H3 (Merck, 05-806, rabbit polyclonal, 1:1000), anti-GFP (Nakalai Tesque, 04404-84, rat monoclonal, 1:1000), and anti-Ki67 (Abcam, ab15589, mouse monoclonal, 1:1000) antibodies. After washing, the sections were incubated with secondary antibodies, including Alexa-Fluor 488, 594 or 633-conjugated anti-rabbit and anti-rat antibodies (Life Technologies, 1:500). Fluorescent images were captured with fluorescent microscopes (SZX7, OLYMPUS; BX51, OLYMPUS) equipped with a cooled CCD camera (DP80, OLYMPUS) and a laser confocal microscope (FV1000D, Olympus). All captured images were processed with cell Sense standard (v.1.17, OLYMPUS), FV10-ASW (v4.2, OLYMPUS), ImageJ (Image analysis in Java 1. 45 s, NIH), and Adobe Photoshop (v22.42, Adobe Systems).

### Coimmunoprecipitation and western blotting

Cultured chick neural progenitors transfected with pCMV-Dll1-HA and pCMV-ubiquitin-Myc were lysed in RIPA buffer (20 mM Tris, 150 mM NaCl, 1 mM EDTA, 1% Nonidet P40, 1% SDS, 0.1% deoxycholate, 1 mM NaF and protein inhibitor). Samples were incubated with anti-HA agarose (Pierce HA Tag IP/Co-IP application set, Thermo Fisher Scientific) at 4 °C overnight. For western blotting, proteins were transferred to PVDF membranes, which were then incubated with anti-c Myc (MBL, 562, rabbit polyclonal, 1:1000) or anti-HA (BioLegend, MMS-101R, mouse monoclonal, 1:1000), followed by HRP-conjugated anti-mouse or anti-rabbit IgG (Vector Laboratories, USA), developed with Chemi-Lumi One Super (Nacalai Tesque, Japan) and analyzed with a luminescent image analyzer (LAS-2000, Fujifilm, Japan). To detect NICD and Dll1 by western blotting, an anti-NICD (Merck, 07-1232, rabbit polyclonal, 1:1000), and ant-Dll1 antibody (R&D systems, AF5026, sheep polyclonal, 1:1000) was used as the primary antibody. Anti-α-tubulin antibody (Abcam, ab9267, rat monoclonal, 1:1000) and β-actin antibody (Abcam, ab8277, rabbit polyclonal, 1:1000) were used as the primary antibody for the internal control of western blotting.

### Whole-brain culture

The whole-brain culture was performed following a modified version of a protocol form a previous report^54^. Isolated mouse brains were cultured in rotating culture bottles filled with DMEM supplemented with fetal bovine serum (10%) and antibiotics (penicillin/streptomycin). The whole-brain culture was performed by using a whole-embryo culture system (IKEMOTO Sci Inc.) supplied with 60% O_2_ at 30 °C or 37 °C for 24 h.

### *In ovo* electroporation

The *in ovo* electroporation of the developing chick and turtle pallium was performed according to a previous study^34,55^. Briefly, a small window was opened in the shell of an incubated egg, and a small amount of DNA solution (less than 0.05 µL) was injected into the lateral ventricle with a small glass needle. Needle-type electrodes (CUY200S, BEX) were placed on the embryonic head, and square electric pulses (28 V for 50 ms, 2 or 3 times) were applied to target the pallium with a pulse generator (CUY21 EDITII, BEX). After electroporation, the extraembryonic cavity was filled with sterilized Hank’s balanced salt solution (HBSS) containing antibiotics (penicillin/streptomycin and gentamycin), and the window was sealed. The electroporated embryos were incubated in an incubator at 30 °C or 37 °C for 24 h. To examine S-phase re-entry in the developing chick pallium, we first electroporated the GFP expression vector in the pallium, by which proliferating ventricular progenitors were efficiently labeled^56^, then we administrated the EdU solution into the lateral ventricle 2 h before fixation.

### *In utero* electroporation

*In utero* electroporation was performed according to previously described^57^. Briefly, a pregnant mouse was anesthetized with isoflurane, and an incision was made in the abdominal wall to expose the uterine horns. A small amount of DNA solution (0.3–0.5 µL) was injected into the lateral ventricle of each embryo, and square pulses (35 V, 50 ms, four times) were applied to the embryos with a tweezer-type electrode (CUY650P3, BEX) connected to a pulse generator (BEX).

### RNA-sequencing data analysis

Total RNA was extracted from cultured chick neural progenitors according to the manufacturer’s protocols (Novogene). RNA quality was assessed using a NanoPhotometer spectrophotometer (IMPLEN, USA). The cDNA library was constructed using a NEBNext Ultra RNA Library Prep Kit for Illumina and sequenced on an Illumina HiSeq platform and 125 bp/15 bp paired-end reads were generated. The sequence data were mapped to a reference genome sequence [*Galgal4*: https://www.ncbi.nlm.nih.gov/assembly/GCF_000002315.3/).

### Analysis of phospholipid compositions

Total lipids were extracted from a cultured chick, mouse, and turtle neural progenitors using the Bligh & Dyer method^58^ and dissolved in chloroform. Phospholipid contents were determined by inorganic phosphate quantification^59^. The analysis of phospholipids was performed with a high-performance liquid chromatography system (LC-30AD, Shimadzu, Kyoto, Japan) coupled to a triple quadrupole mass spectrometer (LC-MS-8040, Shimadzu) equipped with an electrospray source^60^. Separation was performed in a Kinetex C8 column (2.6 μm, 2.1 × 150 mm) (Phenomenex, Torrance, CA, USA) with a binary mobile phase with the following composition: 10 mM ammonium formate in water (mobile phase A) and 10 mM ammonium formate in 2-propanol/acetonitrile/water (45:45:10, v/v/v) (mobile phase B). The pump controlling the gradient of mobile phase B was programmed as follows: an initial isocratic flow at 20% B for 1 min, a linear increase to 40% B for 1 min, an increase to 92.5% B using a curved gradient for 23 min, a linear increase to 100% B for 1 min, and hold at 100% B for 4 min. The total flow rate was 0.3 ml/min, the column temperature was 45 °C, and the sample temperature was 4 °C. The spectrometer parameters were as follows: nebulizer gas flow 2 L/min, drying gas flow 15 L/min, interface voltage 4.5 kV, DL temperature 250 °C, and heat block temperature 400 °C. The multiple reaction monitoring transition was [M + H]^+^ → [184.1]^+^ for PC and [M + H]^+^ → [M + H – 141.0]^+^ for PE. The fatty acid composition of PC and PE was determined via the product ion scan analysis of [M + HCOO]^–^ and [M − H]^–^ as precursor ions, respectively. PC (12:0–13:0) and PE (12:0–13:0) (Avanti Polar Lipids, AL, USA) were used as internal standards.

### Quantitative real-time PCR

Total RNA was extracted from cultured mouse neural progenitors with an RNeasy Mini Kit (Qiagen), and cDNA was synthesized using random primers with reverse transcriptase (ReverTra Ace, TOYOBO). qPCR was performed on a Light Cycler Nano (Roche, Switzerland) with THUNDERBIRD SYBR qPCR Mix (TOYOBO) according to the manufacturer’s protocols. Gene expression levels were normalized to those of β-actin amplification. Primer sequences for qPCR were designed by primer-BLAST (NCBI). The primer sequences were as follows: 5’-ACGTAGTCCCACCTGCCTATG-3’ and 5’-ACAGGTGCCCTGATTGTAGCA-3’ (mouse Notch1: ENSMUSG00000026923), 5’-CAACCAAGACCTGAACTACT-3’ and 5’-CACTCATCTACTTCCAGCTC-3’ (mouse Dll1: ENSMUG00000014773). qPCR was carried out on four biologically independent samples with two technical replicates.

### Preparation of siRNAs

siRNAs that target mouse or chick *Psd1* mRNA was designed with the publicly available software (siDirect v2.0) and chemically synthesized (BEX). The sense and antisense strand sequences of siRNAs were as follows: 5’-AUAAAUGAUUUUACUUUUCCA-3’ and 5’-GAAAAGUAAAAUCAUUUAUAU-3’ (mouse_*Psd1*siRNA1), 5’-GAGUAUUUGGCCUAUAUUUAC-3’ and 5’- AAAUAUAGGCCAAAUACUCUA-3’ (chick_*Psd1*siRNA1), 5’-GUAAAAGGGGUUACUUAUUCU-3’ and 5’-AAUAAGUAACCCCUUUUACUU-3’ (chick_*Psd1*siRNA4), 5’-GAAACAUGGCUUUUUCUCUUU-3’ and 5’-AGAGAAAAAGCCAUGUUUCCA-3’ (chick_*Psd1*siRNA5), 5’ CAAAUUCCACCUUAAAGCUGG-3’ and 5’- AGCUUUAAGGUGGAAUUUGAA-3’ (chick_*Psd1*siRNA6). A siRNA with no homologous sequences in eukaryotes was used as a negative control (Universal negative control siRNA, NEGS 210210, Nippon gene). To validate the knockdown efficiency, each siRNA was introduced into HEK293T cells together with the pGL3-promoter-m*Psd1*–3’UTR or pCMV-chick Psd1-Myc vector and dual-luciferase assays or western blotting were performed. After 24 h, chick Psd1 protein was quantified by western blotting. The western blotting data of chick_*Psd1*siRNA1, 4, 5, and 6 are available from Source Data Files.

### Incubation of chick neural progenitors with a PE-binding peptide

The application of a PE-binding peptide (SA-Ro) to cultured chick neural progenitors was performed as previously described^32^. Briefly, isolated chick neural progenitors were cultured on laminin-coated glass slides (Lab-Tek Chamber Slides 158599, Nunc) at 30 °C or 37 °C for 24 h and incubated with PBS containing 0.1% BSA followed by SA-Ro (5 µg mL^−1^) for 2 h. After fixation with 4% PFA and extensive washing with PBS, chick neural progenitors were incubated with FITC-conjugated biotin (1:200) for 1 h. To assess Notch activity by trapping cell surface PE, neurospheres transfected with p4xCSL-luc and pRL-SV40 were incubated with SA-Ro (5 µg mL^−1^) for 12 h, and luciferase activity was examined.

### Statistical analysis

For statistical analysis, three to six independent samples from each experimental group were compared. Comparisons between experimental groups were performed using Microsoft Excel (v16.54, Microsoft) and Prism 9 (v9.1.2, GraphPad Software Inc). All data are presented as the mean + SD. Statistical significance was determined using the two-tailed unpaired Student’s *t-*test, ordinary one-way ANOVA with Tukey’s multiple comparisons test, or two-way ANOVA with Sidak’s multiple comparison test. For multiple comparisons, all *p*-values were adjusted. In the data obtained from Sox2-negative cells among GFP-positive cells, the matching of observed numbers and expected values was analyzed by using the chi-square test. Differential gene expression analyses of RNA-sequencing data between the 30 °C and 37 °C conditions were performed using the DESeq R package (1.18.0, Bioconductor), which provides statistical routines for determining differential expression in digital gene expression data using a model based on the negative binomial distribution. The resulting *p*- values were adjusted using Benjamin–Hochberg’s approach for controlling the false discovery rate. Gene Ontology (GO) enrichment analysis is performed with GOseq R package (Release 2.12, Bioconductor) based on the Wallenius non-central hypergeometric distribution for multiple comparisons. KEGG enrichment analysis based on the database https://www.genome.jp/kegg/ was performed using KOBAS software (v2.0, http://kobas.cbi.pku.edu.cn) and test the enrichment of differential expression genes in KEGG pathways. *P*-values were estimated by a hypergeometric test, and *p* < 0.05 were considered statistically significant. Q value is the adjusted *p*-values after multiple hypothesis testing.

### Reporting summary

Further information on research design is available in the Nature Research Reporting Summary linked to this article.

## Supplementary information

Supplementary Information

Reporting Summary

## Data Availability

The list of genes showing temperature-dependent expression in chick neural progenitors is available from Mendeley Data (10.17632/9zxt47grjf.2; https://data.mendeley.com/datasets/9zxt47grjf/1). The raw data of RNA sequencing derived from chick neural progenitors have been deposited to the DDBJ database (DRA012953). Sample information of RNA sequencing is also available on European Nucleotide Archive (https://www.ebi.ac.uk/ena/browser/view/DRA012953). All data generated in this study have been deposited to Mendeley Data (10.17632/j2t6c343hg.1; https://data.mendeley.com/datasets/j2t6c343hg/1). Source data are provided with this paper.

## Source data

Source Data
