# Supplementary material for: Temperature sensitivity of Notch signaling underlies species-specific developmental plasticity and robustness in amniote brains
Source: Nat Commun. 2022 Jan 10;13:96. doi: 10.1038/s41467-021-27707-5 (PMC8748702; doi:10.1038/s41467-021-27707-5)
Supplement: Supplementary file 1 — Supplementary Information [file 41467_2021_27707_MOESM1_ESM.pdf]

Supplementary Information

Temperature sensitivity of Notch signaling underlies species-specific developmental plasticity and robustness in amniote brains

Tadashi Nomura[1]*, Kohjiro Nagao[2,3], Ryo Shirai[4,5], Hitoshi Gotoh[1], Masato Umeda[2,6], Katsuhiko Ono[1]

[1]Developmental Neurobiology, Kyoto Prefectural University of Medicine, INAMORI Memorial Building, 1-5 Shimogamo-Hangi cho, Sakyo-ku, Kyoto 606-0823, Japan
[2]Department of Synthetic Chemistry and Biotechnology, Graduate School of Engineering, Kyoto University, Katsura, Nishikyo-ku, Kyoto 615-8510, Japan.
[3]Department of Biophysical Chemistry, Kyoto Pharmaceutical University, 5 Misasaginakauchi-cho, Yamashina-ku, Kyoto 6078414, Japan.
[4]School of Medicine, Niigata University, 757 Ichibancho, Asahimachi-dori, Chuo Ward, Niigata City 951-8510, Japan.
[5]Japanese Red Cross Society Kyoto Daini Hospital, 355-5 Haru-obi cho, Marutamachi-noboru, Kamaza-dori, Kamigyo-ku, Kyoto 602-8026, Japan
[6]HOLO BIO Co., Ltd. 1-36 Goryo Ohara, Nichikyo-ku, Kyoto 615-8245, Japan.

*email: tadnom@koto.kpu-m.ac.jp

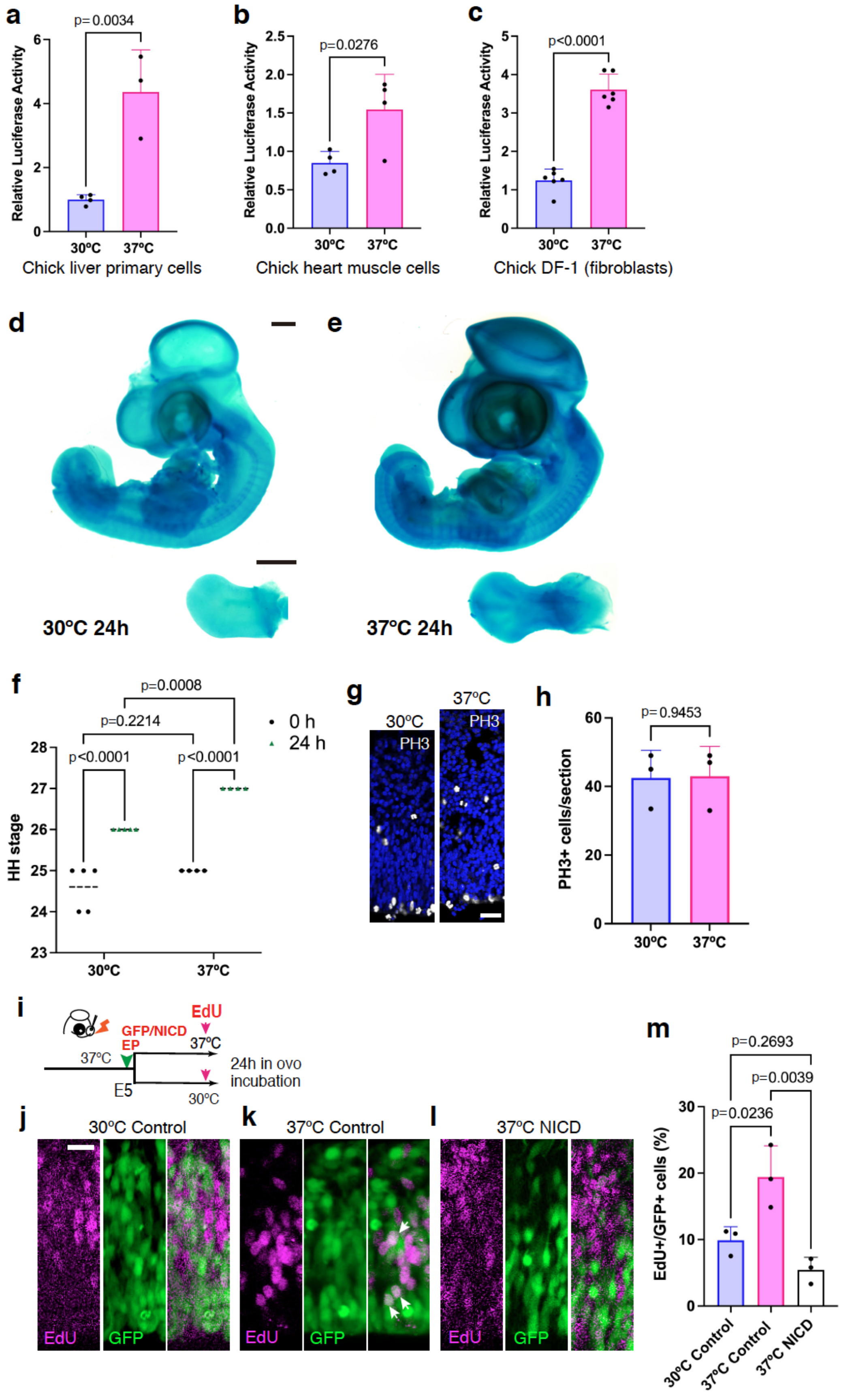
a
p=0.0034
Relative Luciferase Activity
30ºC
37ºC
Chick liver primary cells
b
p=0.0276
Relative Luciferase Activity
30ºC
37ºC
Chick heart muscle cells
c
p<0.0001
Relative Luciferase Activity
30ºC
37ºC
Chick DF-1 (fibroblasts)
d
30ºC 24h
e
37ºC 24h
f
p=0.0008
p=0.2214
p<0.0001
p<0.0001
0 h
24 h
HH stage
30ºC
37ºC
g
30ºC
37ºC
PH3
PH3
h
p=0.9453
PH3+ cells/section
30ºC
37ºC
i
GFP/NICD
EP
EdU
37ºC
37ºC
E5
30ºC
24h in ovo
incubation
j
30ºC Control
k
37ºC Control
l
37ºC NICD
EdU
GFP
m
p=0.2693
p=0.0039
p=0.0236
EdU+/GFP+ cells (%)
30ºC Control
37ºC Control
37ºC NICD

Fig. S1 Hypothermia-dependent changes in Notch signaling and cellular dynamics in the developing chick embryo

(**a**-**c**) Notch reporter activity in chick liver primary cells (**a**), heart muscle cells (**b**), and DF-1 fibroblast cells (**c**) cultured at 30 °C or 37 °C [mean + SD, *n*=4 and 3 biologically independent samples in 30 °C and 37 °C (**a**), *n*=4 biologically independent samples in each group (**b**), and *n*=6 biologically independent samples in each group (**c**)]. (**d**, **e**) Alcian blue staining of chick embryos incubated at 30 °C (**d**) or 37 °C (**e**) for 24 hours. (**f**) HH stages of chick embryos at 0 hours and 24 hours of incubation at 30°C or 37 °C (*n*= 5 and 4 biologically independent samples in 30 °C and 37 °C groups, dashed lines indicate mean values). (**g**) Distribution of phospho-histone H3 (PH3)-positive cells in the developing chick pallium incubated at 30 °C or 37 °C for 24 hours. (**h**) The number of PH3-positive cells in the developing chick pallium at different temperatures (*n*=3 biologically independent samples in each group). (**i**) Schematic drawing on the analysis of S-phase re-entry in the developing chick pallium. (**j**-**l**) Distribution of GFP-positive cells and/or EdU-labeled cells 30 °C control (**j**), 37°C control (**k**), and 37 °C NICD overexpressed samples (**l**). (**m**) The proportion of EdU-positive cells among GFP-labeled cells in controls (30 °C and 37 °C) and NICD overexpressed samples (mean + SD, *n*=3 biologically independent samples in each group). Two-sided, unpaired *t*-test for **a-c**, **h**; ordinary two-way or one-way ANOVA for **f, m** (p values were adjusted by Tukey or Sidak test for multiple comparisons). Scale bars: 500μm (**d**, **e**); 25μm (**g, j**).

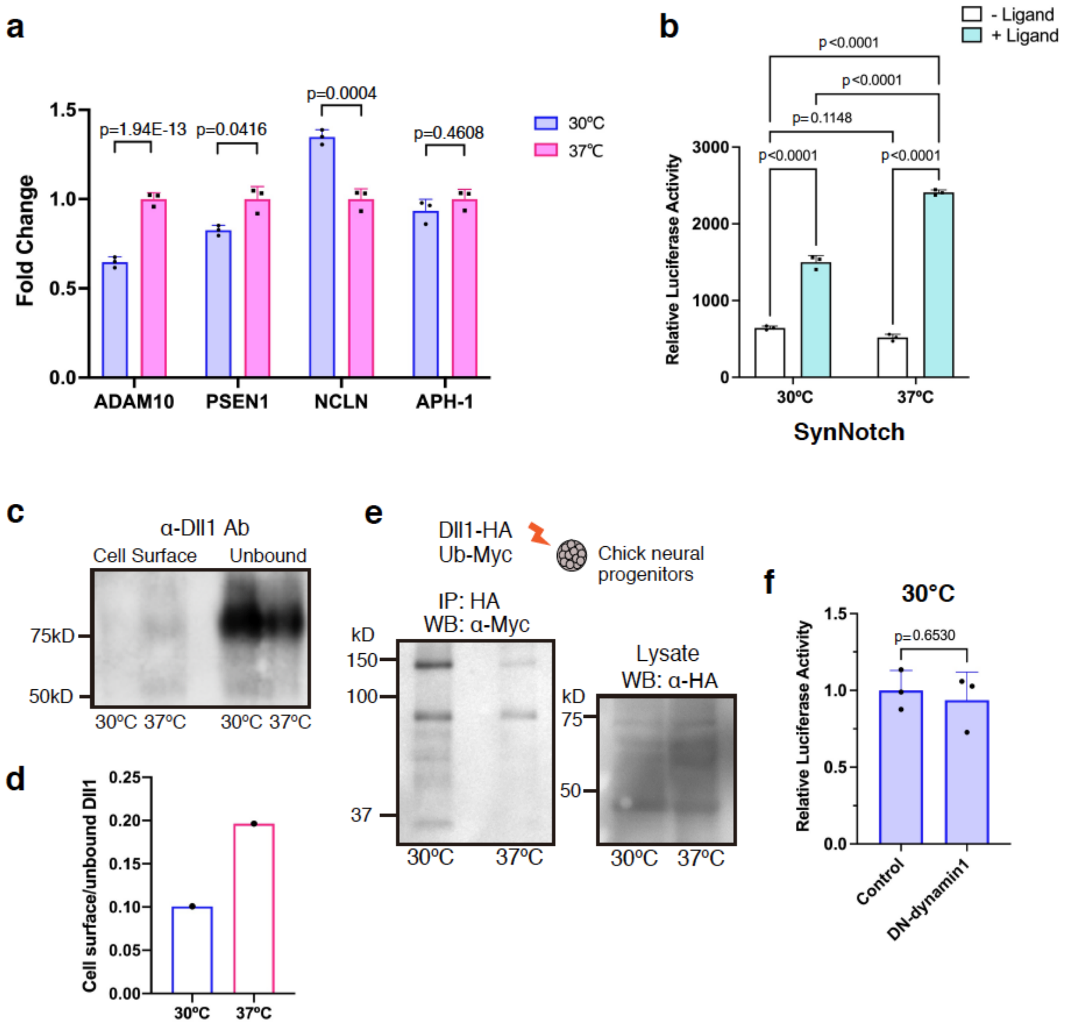

Fig. S2 The contribution of ligand internalization to hypothermia-dependent Notch activity

(**a**) Relative expression levels of genes encoding ADAM10 or γ-secretase components (PSEN1, NCLN, and APH-1) in chick neural progenitors cultured at 30 °C or 37 °C (mean + SD, *n*=3 biologically independent samples in each group). (**b**) Luciferase activity of the SynNotch receptor with/without ligands (GFP nanobody) in chick neural progenitors cultured at different temperatures (mean + SD, *n*=3 biologically independent samples in each group). (**c**) Immunoprecipitation of the cell surface and internal Dll1 in chick neural progenitors cultured at different temperatures. Dll1 in biotinylated and unbound proteins was detected using an anti-Dll1 antibody. (**d**) The ratio of cell surface/unbound Dll1 in neural progenitors cultured at 30 °C or 37 °C. The graph is conducted from the data of Fig. S2c (*n*=1 sample in each temperature group) (**e**) Immunoprecipitation of ubiquitylated Dll1 in chick neural progenitors cultured at 30 °C or 37 °C. (**f**) Notch

reporter activity in control and dominant negative-dynamin1 (DN-dynamin 1) introduced chick neural progenitors at 30 °C (mean + SD, *n*=3 biologically independent samples in each group). Two-sided, negative bimodal distribution for **a** (p values were adjusted for multiple comparisons by using Benjamin & Hochberg method), ordinary two-way ANOVA for **b** (p values were adjusted by Sidak test for multiple comparisons), two-sided, unpaired *t*-test for **f**.

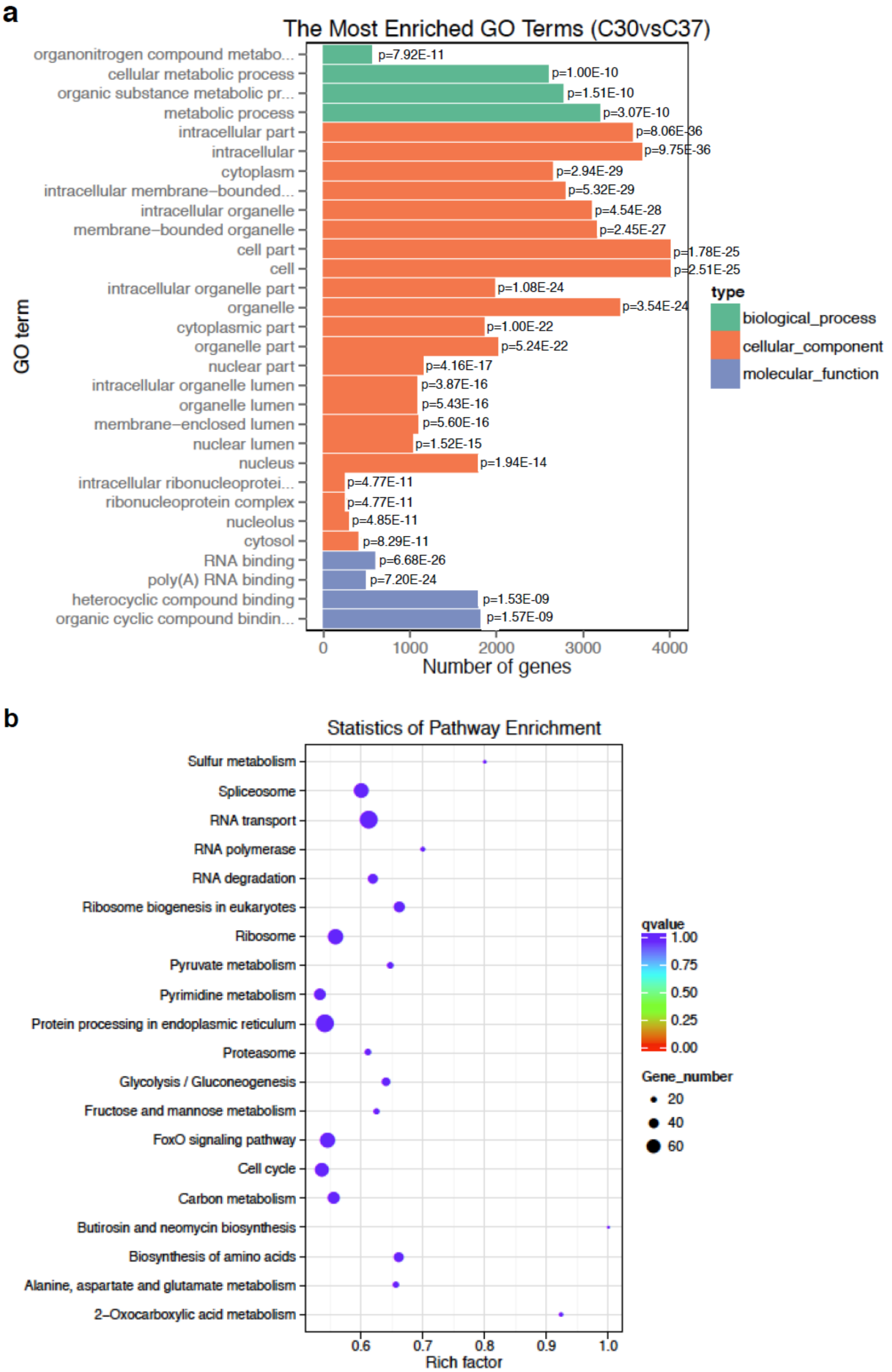

The KEGG Enrichment Scatter Plot

Fig. S3 GO enrichment analysis of differentially expressed genes in chick neural progenitors cultured by temperature variations

(**a**) Bar chart showing top 30 most enriched GO terms in differentially expressed genes (DEGs) between 30°C and 37°C. Different colors represent biological process, cellular components, and molecular functions. (**b**) KEGG enrichment scattered plot of DEGs under different temperatures. Top 20 most significant enriched pathways are represented. Rich factor is the ratio of DEGs counts to each pathway in the annotated gene counts. The most enriched KEGG pathways with high rich factors are 1) RNA transport, 2) ribosome biogenesis in eukaryotes, 3) spliceosome, and 4) biosynthesis of amino acids. P values in (**a**) were estimated and corrected by using two-sided, Wallenius non-central hyper-geometric distribution for multiple comparisons.

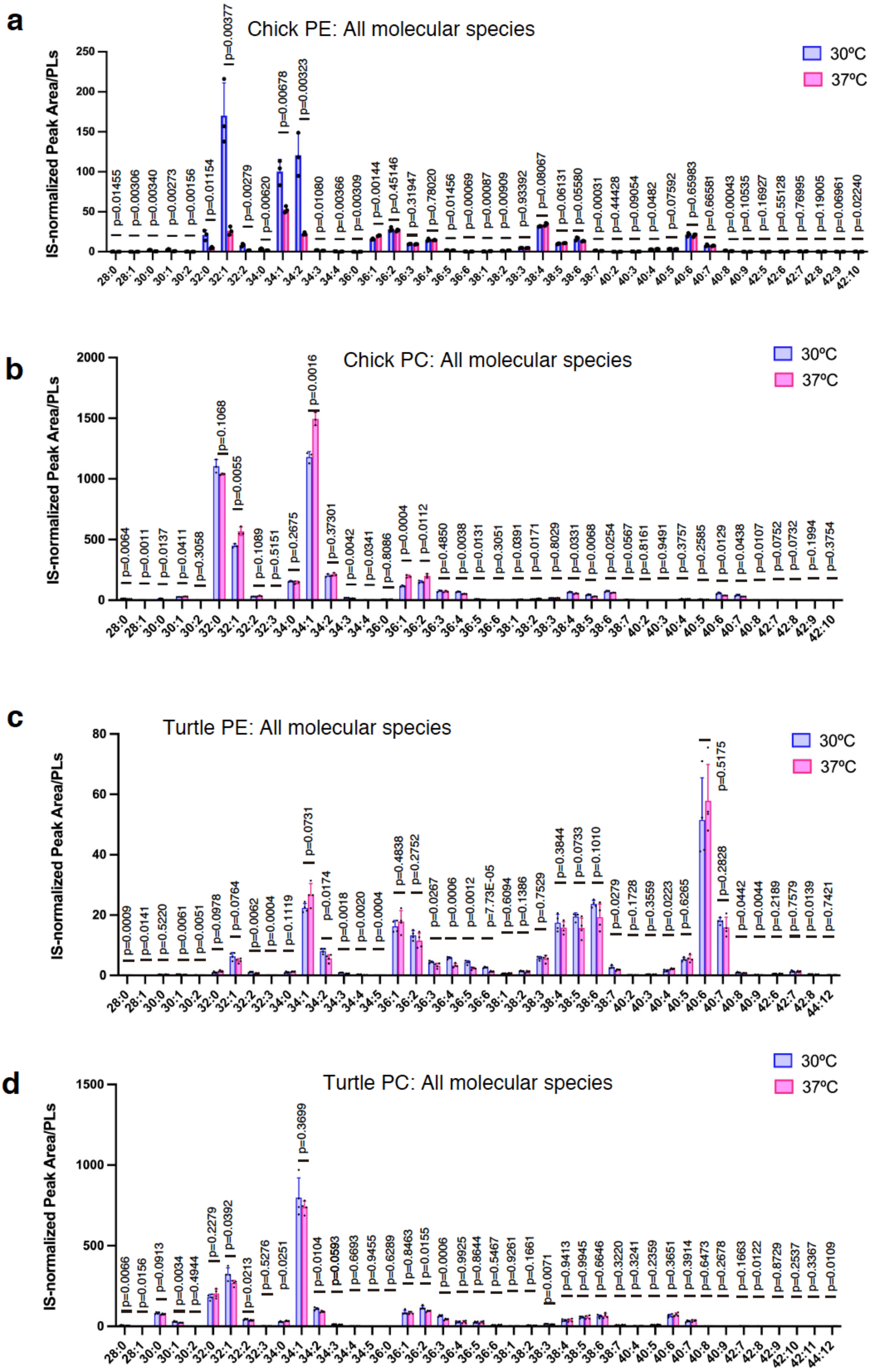
a
Chick PE: All molecular species
b
Chick PC: All molecular species
c
Turtle PE: All molecular species
d
Turtle PC: All molecular species
IS-normalized Peak Area/PLs
30ºC
37ºC

Fig. S4 Temperature-dependent changes in phospholipids in chick and turtle neural progenitors

(**a**, **b**) Amounts of all PE (**a**) and PC (**b**) molecules in chick neural progenitors cultured at 30 °C or 37 °C (mean + SD, n=3 biologically independent samples in each group) (**c**, **d**) Amounts of all PE (**c**) and PC (**d**) molecules in turtle neural progenitors cultured at 30 °C or 37 °C (mean + SD, n=4 biologically independent samples in each group). P values were estimated by Two-sided, unpaired *t*-test.

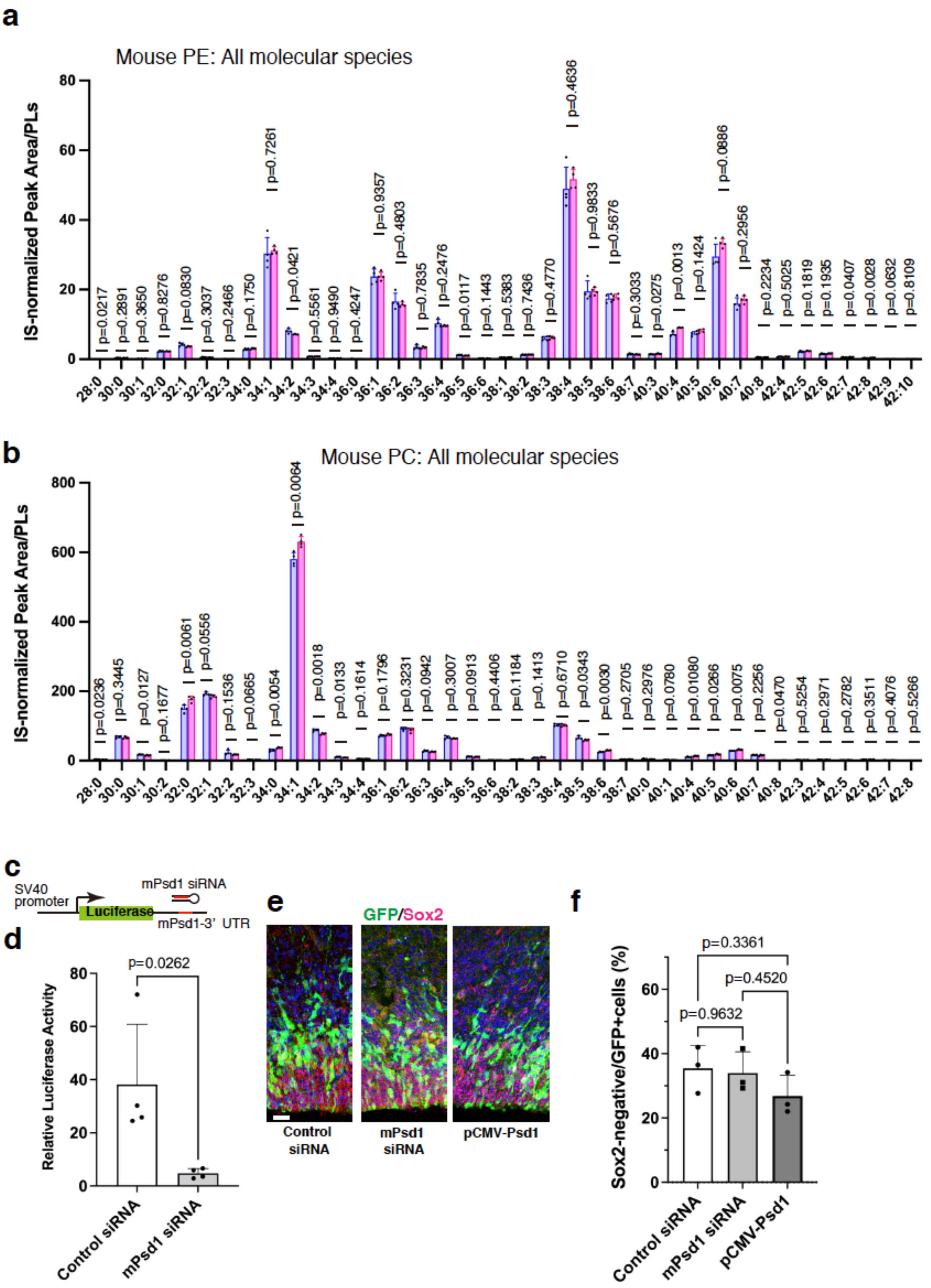
a
Mouse PE: All molecular species
IS-normalized Peak Area/PLs
80
60
40
20
0
p=0.0217
p=0.2891
p=0.3650
p=0.8276
p=0.0830
p=0.3037
p=0.2466
p=0.1750
p=0.7261
p=0.0421
p=0.5561
p=0.9490
p=0.4247
p=0.9357
p=0.4803
p=0.7835
p=0.2476
p=0.0117
p=0.1443
p=0.5383
p=0.7436
p=0.4770
p=0.4636
p=0.9833
p=0.5676
p=0.3033
p=0.0275
p=0.0013
p=0.1424
p=0.0886
p=0.2956
p=0.2234
p=0.5025
p=0.1819
p=0.1935
p=0.0407
p=0.0028
p=0.0632
p=0.8109
28:0 30:0 30:1 32:0 32:1 32:2 32:3 34:0 34:1 34:2 34:3 34:4 36:0 36:1 36:2 36:3 36:4 36:5 36:6 38:1 38:2 38:3 38:4 38:5 38:6 38:7 40:3 40:4 40:5 40:6 40:7 40:8 42:4 42:5 42:6 42:7 42:8 42:9 42:10
b
Mouse PC: All molecular species
IS-normalized Peak Area/PLs
800
600
400
200
0
p=0.0236
p=0.3445
p=0.0127
p=0.1677
p=0.0061
p=0.0556
p=0.1536
p=0.0665
p=0.0054
p=0.0064
p=0.0018
p=0.0133
p=0.1614
p=0.1796
p=0.3231
p=0.0942
p=0.3007
p=0.0913
p=0.4406
p=0.1184
p=0.1413
p=0.6710
p=0.0343
p=0.0030
p=0.2705
p=0.2976
p=0.0780
p=0.01080
p=0.0266
p=0.0075
p=0.2256
p=0.0470
p=0.5254
p=0.2971
p=0.2782
p=0.3511
p=0.4076
p=0.5266
28:0 30:0 30:1 30:2 32:0 32:1 32:2 32:3 34:0 34:1 34:2 34:3 34:4 36:1 36:2 36:3 36:4 36:5 36:6 38:2 38:3 38:4 38:5 38:6 38:7 40:0 40:1 40:4 40:5 40:6 40:7 40:8 42:3 42:4 42:5 42:6 42:7 42:8
c
SV40 promoter
Luciferase
mPsd1 siRNA
mPsd1-3' UTR
d
Relative Luciferase Activity
p=0.0262
80
60
40
20
0
Control siRNA
mPsd1 siRNA
e
GFP/Sox2
Control siRNA
mPsd1 siRNA
pCMV-Psd1
f
Sox2-negative/GFP+cells (%)
p=0.3361
p=0.4520
p=0.9632
60
40
20
0
Control siRNA
mPsd1 siRNA
pCMV-Psd1

Fig. S5 Temperature-dependent changes in phospholipids in mouse neural progenitors

(**a**, **b**) Amounts of all PE (**a**) and PC (**b**) molecules in mouse neural progenitors cultured at 30 °C or 37 °C (mean + SD, *n*=4 biologically independent samples in each group). (**c**, **d**) Luciferase reporter-based validation of mouse Psd1 (mPsd1) siRNA. (**c**) Reporter vector containing siRNA target sequence (mPsd1-3'UTR) was co-transfected with control siRNA or mPsd1 siRNA. (**d**) mPsd1 siRNA significantly reduced luciferase activity compared to control siRNA (mean + SD, *n*=4 biologically independent samples in each group). (**e**) Distributions of GFP-positive cells in the developing mouse neocortex that were co-transfected with control siRNA or *mPsd1* siRNA. (**f**) The proportion of Sox2-negative cells among GFP-labeled cells in the neocortex transfected with control siRNA, *mPsd1* siRNA, and *mPsd1* (mean + SD, *n*=3 biologically independent samples in each group). Two-sided, unpaired *t*-test for **a**, **b**, **d**, ordinary one-way ANOVA for **f** (p values were adjusted by Tukey for multiple comparisons). Scale bars: 20µm.

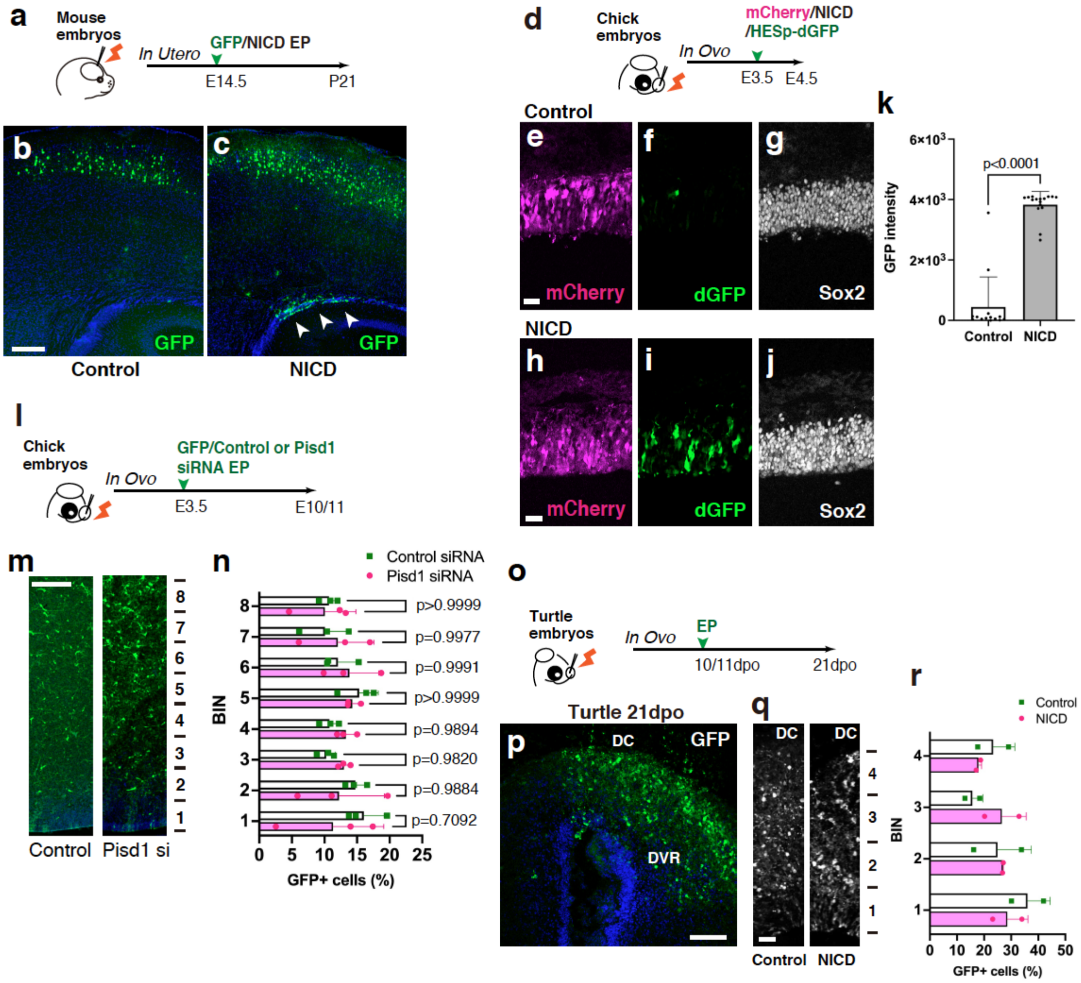

Fig. S6 The effects of increased Notch signaling or knock down of Psd1 in the developing amniote pallium

(**a**-**c**) Long-term effects of NICD in the developing mouse neocortex. (**a**) A schematic drawing of *in utero* electroporation and phenotype analysis. (**b**, **c**) Distribution of GFP-positive cells in postnatal day 21 (P21) mouse neocortex transfected with GFP control (**b**) or NICD (**c**) expression vector. Arrowheads indicate abnormal cell accumulation in the subventricular zone of a postnatal mouse. (**d**-**k**) Monitoring of NICD-dependent Hes1 expression in the developing chick pallium by introducing Hes1p-GFPd2 reporter vector with/without NICD expression vector. (**d**) A schematic drawing of *in ovo* electroporation and phenotype analysis. A mCherry expression vector was co-transfected to visualize electroporated cells. (**e**-**j**) Expression patterns of dGFP driven by Hes1 promoter activity in control (**e**-**g**) and NICD overexpressed samples (**h**-**j**). (**k**) GFP fluorescent intensities in control and NICD introduced samples (mean + SD, *n*=14 cells in control and *n*=16 cells in NICD transfected animals). (**l**-**n**) Introduction of chick *Psd1* siRNA into the developing

chick pallium. (**l**) A schematic drawing of *in ovo* electroporation and phenotype analysis. (**m**) Distribution of GFP-positive cells in the pallium of control siRNA and *cPsd1* siRNA introduced samples. (**n**) Quantification of GFP-positive cells in control siRNA and *cPsd1* siRNA overexpressed pallium (mean + SD, *n*=3 postnatal 21-day in each group). (**o-r**) Introduction of NICD into the developing turtle dorsal pallium. (**o**) A schematic drawing of *in ovo* electroporation and phenotype analysis. (**p, q**) Distribution of GFP-positive cells in the dorsal cortex of control and NICD introduced samples. (**r**) Quantification of GFP-positive cells in control and NICD overexpressed cortex (*n*=2 biologically independent samples in each group). Two-sided, unpaired *t*-test for **k**, two-way ANOVA for **n** (p values were adjusted by Sidak test). Scale bars: 100 µm (**b**, **m**, **p**); 20 µm (**h**, **q**)
