# Supplementary material for: Temperature sensitivity of Notch signaling underlies species-specific developmental plasticity and robustness in amniote brains
Source: Nat Commun. 2022 Jan 10;13:96. doi: 10.1038/s41467-021-27707-5 (PMC8748702; doi:10.1038/s41467-021-27707-5)
Supplement: Supplementary file 2 — Reporting Summary [file 41467_2021_27707_MOESM2_ESM.pdf]

nature portfolio

Corresponding author(s): Tadashi Nomura

Last updated by author(s): 2021-10-29

Reporting Summary

Nature Portfolio wishes to improve the reproducibility of the work that we publish. This form provides structure for consistency and transparency in reporting. For further information on Nature Portfolio policies, see our Editorial Policies and the Editorial Policy Checklist.

Statistics

For all statistical analyses, confirm that the following items are present in the figure legend, table legend, main text, or Methods section.

n/a	Confirmed	
☐	☒	The exact sample size (n) for each experimental group/condition, given as a discrete number and unit of measurement
☐	☒	A statement on whether measurements were taken from distinct samples or whether the same sample was measured repeatedly
☐	☒	The statistical test(s) used AND whether they are one- or two-sided<br>*Only common tests should be described solely by name; describe more complex techniques in the Methods section.*
☒	☐	A description of all covariates tested
☐	☒	A description of any assumptions or corrections, such as tests of normality and adjustment for multiple comparisons
☐	☒	A full description of the statistical parameters including central tendency (e.g. means) or other basic estimates (e.g. regression coefficient) AND variation (e.g. standard deviation) or associated estimates of uncertainty (e.g. confidence intervals)
☐	☒	For null hypothesis testing, the test statistic (e.g. F, t, r) with confidence intervals, effect sizes, degrees of freedom and P value noted<br>*Give P values as exact values whenever suitable.*
☒	☐	For Bayesian analysis, information on the choice of priors and Markov chain Monte Carlo settings
☒	☐	For hierarchical and complex designs, identification of the appropriate level for tests and full reporting of outcomes
☒	☐	Estimates of effect sizes (e.g. Cohen's d, Pearson's r), indicating how they were calculated

Our web collection on statistics for biologists contains articles on many of the points above.

Software and code

Policy information about availability of computer code

Data collection	cell Sense Standard (v.1.17, OLYMPUS), FV10-ASW (v4.2, OLYMPUS)
Data analysis	Photoshop 2021 (v22.5.1, Adobe), ImageJ (v1.45s, NIH), DESeq R package (v1.18.0, Bioconductor), DOSeq R package (Release 2.12, Bioconductor) Microsoft Excel (v16.54, Microsoft),<br>KOBAS software (v2.0, http://kobas.cbi.pku.edu.cn), Prisim 9 (v9.1.2, Graph Pad Software Inc.)

For manuscripts utilizing custom algorithms or software that are central to the research but not yet described in published literature, software must be made available to editors and reviewers. We strongly encourage code deposition in a community repository (e.g. GitHub). See the Nature Portfolio guidelines for submitting code & software for further information.

Data

Policy information about availability of data

All manuscripts must include a data availability statement. This statement should provide the following information, where applicable:

- Accession codes, unique identifiers, or web links for publicly available datasets
- A description of any restrictions on data availability
- For clinical datasets or third party data, please ensure that the statement adheres to our policy

The list of differential gene expression has been deposited to Mendeley Data (doi: 10.17632/9zxt47grjf.1).
Raw data of RNAseq have been deposited to DDBJ database (DRA012953) and European Nucleotide Archives (DRA012953).
Raw data for each figure have been deposited to Mendelay Data (doi: 10.17632/9zxt47grjf.1)

Field-specific reporting

Please select the one below that is the best fit for your research. If you are not sure, read the appropriate sections before making your selection.

- [X] Life sciences
- [ ] Behavioural & social sciences
- [ ] Ecological, evolutionary & environmental sciences

For a reference copy of the document with all sections, see nature.com/documents/nr-reporting-summary-flat.pdf

Life sciences study design

All studies must disclose on these points even when the disclosure is negative.

Sample size	Sample size were estimated on the basis of previous studies using similar methods and analyses (Shimizu et al. 2014, doi: 10.1016/j.cell.2014.03.050; Whiteman et al. 2013, doi: 10.1074/jbc.M112.428854) that provide sufficient statistical power for analysis. Each replicate contained a large amount of cell information.
Data exclusions	No samples/data were excluded.
Replication	Each experiment was reproduced with at least three biologically independendet replicates. The data of Figure S6r was performed with two biological independent samples. The number of samples and statements were described in the figure legends.
Randomization	All cell populations and animals used for experiments were randomly allocated.
Blinding	In luciferase assay, RNAseq, western blotting, and lipid cmponent measurement, the invesigators were not blinded during data collection and analysis, becauce these analyses were done in automated fashions eliminating human bias. For histological data collection, we randomely collected multiple fields per goup/section and caluculated the number of labeled cells per fields. Data collection and analysis were done by distinct persons in order to eliminate unintended human bias.

Behavioural & social sciences study design

All studies must disclose on these points even when the disclosure is negative.

Study description	
Research sample	
Sampling strategy	
Data collection	
Timing	
Data exclusions	
Non-participation	
Randomization	

Ecological, evolutionary & environmental sciences study design

All studies must disclose on these points even when the disclosure is negative.

Study description	
Research sample	

Reporting for specific materials, systems and methods

We require information from authors about some types of materials, experimental systems and methods used in many studies. Here, indicate whether each material, system or method listed is relevant to your study. If you are not sure if a list item applies to your research, read the appropriate section before selecting a response.

Materials & experimental systems

n/a	Involved in the study	
☐	☒	Antibodies
☐	☒	Eukaryotic cell lines
☒	☐	Palaeontology and archaeology
☐	☒	Animals and other organisms
☒	☐	Human research participants
☒	☐	Clinical data
☒	☐	Dual use research of concern

Methods

n/a	Involved in the study	
☒	☐	ChIP-seq
☒	☐	Flow cytometry
☒	☐	MRI-based neuroimaging

Antibodies

Antibodies used

Primary antibodies used for immunohistochemstry:
Sox2, Abcam, ab97959, Polyclonal rabbit IgG, Lot: GR293487-1, Dilution: 1:1000
PH3, Merck (Millipore), 05-806, Polyclonal rabbit IgG, Lot: 2554900, Dilution: 1:1000
GFP, Nacalai Tesque, 04404-84, Monoclonal rat IgG, Lot: M7R1706, Dilution: 1:1000
Ki67, Abcam, ab15589, Monoclonal mouse IgG, Lot: GR250214-1, Dilution: 1:1000

Primary antibodies used for western blotting and immunoprecipitation
Notch1 (NICD), Merck (Millipore), 07-1232, Polyclonal rabbit IgG, Lot: 07-1232 Dilution: 1:1000.
Dll1, R&D Systems, AF5026, Polyclonal sheep IgG, Lot: YXZ0318101, Dilution: 1:2000
Myc, MBL, 562, Polyclonal rabbit IgG, Lot: 055, Dilution: 1:1000
HA, Biolegend (Covance), MMS-101R, Monoclonal mouse IgG, Lot: 1497302, Dilution: 1:1000
β-actin, Abcam, ab8277, Polyclonal rabbit IgG, Lot: GR108484-1, Dilution: 1:1000
α-tubulin, Abcam, ab9267, Monoclonal rat IgG2a, Dilution: 1:1000 (we purchased this antibody at 2003 and lot information of the antibody is not available).

Secondary antiboidies used for immunohistochemstry
Alexa-Fluor 488 Goat anti-rabbit IgG (H+L), Invtrogen, A11034, Lot: 2018207, Dilution: 1:500
Alexa-Fluor 594 Goat anti-rabbit IgG (H+L), Invtrogen, A11012, Lot: 2018240, Dilution: 1:500
Alexa-Fluor 633 Goat anti-rabbit IgG (H+L), Invtrogen, A21070, Lot: 1668691, Dilution: 1:500
Alexa-Fluor 488 Goat anti-rat IgG (H+L), Invtrogen, A11006, Lot: 2005935, Dilution: 1:500
Alexa-Fluor 594 Goat anti-mouset IgG (H+L), Invtrogen, A11032, Lot: 1887003, Dilution: 1:500

Secondary antiboidies used for western blotting and immunoprecipitation
Biotinylated anti-sheep IgG (H+L), Vector laboratories, BA-6000, Lot: ZF0717, Dilution: 1/2000
Biotinylated anti-rabbit IgG (H+L), Vector laboratories, BA-1000, Lot: ZG0122, Dilution: 1/2000
Biotinylated anti-mouse IgG (H+L), Vector laboratories, BA-2020, Lot: ZD0811, Dilution: 1/2000
Biotinylated anti-rat IgG (H+L), Vector laboratories, BA-4000, Lot: ZG0910, Dilution: 1/2000

Validation

Beside initial validation of commercial primary antibodies by manufactures/suppliers, all antibodies utilized in this study were validated in our previous studies as well as other references. Companies provides validation data of each antibody in following websites:
-Anti-Sox2 antibody, abcam https://www.abcam.com/sox2-antibody-ab97959.html
-Anti-PH3 antibody, Merck (Millipore), https://www.merckmillipore.com/JP/en/product/Anti-phospho-Histone-H3-Ser10-Antibody-clone-3H10,MM_NF-05-806?ReferrerURL=https%3A%2F%2Fwww.google.com%2F
-Anti-GFP antibody, Nacalai Tesque, https://www.nacalai.co.jp/ss/ec2/ec-srchdetl.cfm?HP=1&l=EN&lc=1&syohin=0440426&syubetsu=3&catalog=&SiireC=&MakerC=&yoro=&mv=1
-Anti-Ki67 antibody, abcam, https://www.abcam.com/ki67-antibody-ab15580.html
-Anti-Notch1 antibody, Merck (Millipore), https://www.merckmillipore.com/JP/en/product/Anti-Notch-1-Antibody-NT,MM_NF-07-1232?ReferrerURL=https%3A%2F%2Fwww.google.com%2F
-Anti-Dll1 antibody, R&D Systems, https://www.rndsystems.com/products/mouse-rat-dll1-antibody_af3970
-Anti-Myc antibody, MBL, https://ruo.mbl.co.jp/bio/dtl/A/?pcd=562
-Anti-HA antibody, Biolegend, https://www.biolegend.com/ja-jp/products/anti-ha-11-epitope-tag-antibody-11071?GroupID=GROUP26
-Anti-β-actin antibody, Abcam, https://www.abcam.co.jp/beta-actin-antibody-ab8227.html
-Anti-α-tubulin, Abcam (original antibody is not available from abcam. Information of the antibody derived from same clone is provided in the website: https://www.abcam.co.jp/tubulin-antibody-yol134-microtubule-marker-ab6161.html

Eukaryotic cell lines

Policy information about cell lines

Cell line source(s)

HEK293T cells were obtained from RIKEN BRC Cell Bank (RBRC-RCB2202, lot number 010).
DF-1 chick fibroblast cell line was obtained from ATCC (CRL-12203, lot number 60283795).

Authentication	HEK293T cells and DF-1 cells used in this study were validated by STR profiling and species-specific primer tests.
Mycoplasma contamination	Mycoplasma contamination of HEK293T cells and DF-1 cells were checked by fluorescent nuclear staining and PCR, and confirmed negative for contamination.
Commonly misidentified lines (See ICLAC register)	No commonly misidentified cell lines registered in ICLAC were used in this study.

Animals and other organisms

Policy information about studies involving animals; ARRIVE guidelines recommended for reporting animal research

Laboratory animals	Fertilized chicken eggs were purchased from a local Poultly farm (Yamagishi).<br>Fertlized Chinese softshell turtle eggs were purchased from a local breeder (Daiwa-Yoshoku Co., Ltd.).<br>Pregnant female mice (Slc:ICR; 3 months, 17 pregnant animals) were purchased from Japan CLEA Inc.<br>Housing conditions of mice were as follows: 12 hours dark/light cycle, 25 °C, and 60 % humidity.
Wild animals	No wild animals were used.
Field-collected samples	No field-collcted samples were used.
Ethics oversight	All experiments followed relevant guidelines and regulations and were approved by experimental animal committee of Kyoto Prefectural University of Medicine (Dean: Dr. Satoshi Ohtsuka, #M2020-193, M2020-4).

Identify the organization(s) that approved or provided guidance on the study protocol, OR state that no ethical approval or guidance was required and explain why not.

Note that full information on the approval of the study protocol must also be provided in the manuscript.

Human research participants

Policy information about studies involving human research participants

Population characteristics	
Recruitment	
Ethics oversight	

Note that full information on the approval of the study protocol must also be provided in the manuscript.

Clinical data

Policy information about clinical studies

All manuscripts should comply with the ICMJE guidelines for publication of clinical research and a completed CONSORT checklist must be included with all submissions.

Clinical trial registration	
Study protocol	
Data collection	
Outcomes	
